# Small Area Geographic Estimates of Cardiovascular Disease Risk Factors in India

**DOI:** 10.1001/jamanetworkopen.2023.37171

**Published:** 2023-10-12

**Authors:** Soohyeon Ko, Hannah Oh, S. V. Subramanian, Rockli Kim

**Affiliations:** 1 Department of Public Health Sciences, Graduate School of Korea University, Seoul, Republic of Korea; 2 Interdisciplinary Program in Precision Public Health, Korea University, Seoul, Republic of Korea; 3 Division of Health Policy and Management, College of Health Science, Korea University, Seoul, Republic of Korea; 4 Harvard Center for Population and Development Studies, Cambridge, Massachusetts; 5 Department of Social and Behavioral Sciences, Harvard T.H. Chan School of Public Health, Boston, Massachusetts

## Abstract

**Importance:**

With an aging population, India is facing a growing burden of cardiovascular diseases (CVDs). Existing programs on CVD risk factors are mostly based on state and district data, which overlook health disparities within macro units.

**Objective:**

To quantify and geovisualize the extent of small area variability within districts in CVD risk factors (hypertension, diabetes, and obesity) in India.

**Design, Setting, and Participants:**

This cross-sectional study analyzed nationally representative data from the National Family Health Survey 2019-2021, encompassing individuals aged 15 years or older, for hypertension (n = 1 715 895), diabetes (n = 1 807 566), and obesity (n = 776 023). Data analyses were conducted from July 1, 2022, through August 1, 2023.

**Exposures:**

Geographic units consisting of more than 30 000 small areas, 707 districts, and 36 states or Union Territories across India.

**Main Outcomes and Measures:**

For primary outcomes, CVD risk factors, including hypertension, diabetes, and obesity, were considered. Four-level logistic regression models were used to partition the geographic variability in each outcome by state or Union Territory (level 4), district (level 3), and small area (level 2) and compute precision-weighted small area estimates. Spatial distribution of district-wide means, within-district small area variability, and their correlation were estimated.

**Results:**

The final analytic sample consisted of 1 715 895 individuals analyzed for hypertension (mean [SD] age, 39.8 [17.3] years; 921 779 [53.7%] female), 1 807 566 for diabetes (mean [SD] age, 39.5 [17.2] years; 961 977 [53.2%] female), and 776 023 for obesity (mean [SD] age, 30.9 [10.2] years; 678 782 [87.5%] women). Overall, 21.2% of female and 24.1% of male participants had hypertension, 5.0% of female and 5.4% of men had diabetes, and 6.3% of female and 4.0% of male participants had obesity. For female participants, small areas (32.0% for diabetes, 34.5% for obesity, and 56.2% for hypertension) and states (30.0% for hypertension, 46.6% for obesity, and 52.8% for diabetes) accounted for the majority of the total geographic variability, while districts accounted for the least (13.8% for hypertension, 15.2% for diabetes, and 18.9% for obesity). There were moderate to strong positive correlations between district-wide mean and within-district variability (*r* = 0.66 for hypertension, 0.94 for obesity, and 0.96 for diabetes). For hypertension, a significant discordance between district-wide mean and within-district small area variability was found. Results were largely similar for male participants across all categories.

**Conclusions and Relevance:**

This cross-sectional study found a substantial small area variability, suggesting the necessity of precise policy attention specifically to small areas in program formulation and intervention to prevent and manage CVD risk factors. Targeted action on policy-priority districts with high prevalence and substantial inequality is required for accelerating India’s efforts to reduce the burden of noncommunicable diseases.

## Key Points

**Question:**

How much do small areas account for the geographic variability in cardiovascular disease risk factors in India?

**Findings:**

In this cross-sectional study of 1 715 895 individuals analyzed for hypertension, 1 807 566 for diabetes, and 776 023 for obesity, all aged 15 years or older, states and small areas accounted for the largest proportion of geographic variability in cardiovascular disease risk factors in India. In general, districts with a higher mean prevalence tended to have greater inequality among small areas within a district.

**Meaning:**

These findings underscore the importance of considering small areas and prioritizing districts based on high mean and high variability in policy implementation to reduce the burden of cardiovascular disease risk factors in India with greater efficiency.

## Teaser

This cross-sectional study examines the extent of small area variability in major cardiovascular disease risk factors among adults in India.

## Introduction

Noncommunicable diseases (NCDs), such as cardiovascular disease (CVD) and cancer, are the leading causes of premature deaths worldwide,^1^ with the burden disproportionately concentrated in low- and middle-income countries and accounting for more than 85% of annual premature deaths.^2^ Notably, India, the world’s largest and fastest-growing country, witnessed nearly 6 million NCD-related deaths in 2016 alone, constituting 63% of total deaths in the same year.^1^ Despite the escalating burden, health care services for NCDs in India remain inadequately established because of limited resources and competing priorities from other health challenges.^3^ Therefore, it becomes imperative to identify high-priority areas for optimized resource allocation.

Current policies on NCD prevention and control in India have relied on macro geographic and administrative units.^4^ For example, states are principal macro policy units, and districts are subdivisions of states that serve as central policy units for the development, administration, and implementation of programs.^5^ However, prior studies have consistently demonstrated considerable variability across small areas for various development and health outcomes,^6,7,8,9,10,11,12,13,14^ underscoring the significant inequality within any given district and raising concerns about the potential limitations of a one-size-fits-all approach based on district averages. These findings are primarily restricted to child-related issues, such as poor nutrition^8,9,10^ and birth outcomes,^11,12,13^ and there remains limited knowledge about small area variability in CVD risk factors among adults. It is likely that an even greater geographic heterogeneity exists for CVD risk factors, given their strong geographic patterns with sociodemographic factors, as well as accumulated impact of poor access to health care services.^15,16,17^

In this context, our study aims to quantify geographic variability in major CVD risk factors (hypertension, diabetes, and obesity) in India using the recently released National Family Health Survey (NFHS) 2019-2021. First, multilevel analysis was used to partition the geographic variability in CVD risk factors to assess the relative importance of multiple macro and micro units. Second, to underscore the relevance of within-district variability in defining high-priority regions, we estimated and geovisualized district-wide mean, within-district variability, and their correlation.

## Methods

### Data Source and Sample

We used cross-sectional data from the latest NFHS (June 17, 2019, to April 30, 2021), which collected nationally representative samples from 707 districts nested within 28 states and 8 Union Territories of India for the first time.^18^ Unlike previous rounds of the NFHS, the latest survey provides data on hypertension and diabetes for a wider range of age groups. These measurements were collected on all usual household members and visitors aged 15 years or older of the selected households who spent the night in the household. Obesity-related measurements were taken on female participants aged 15 to 49 years and male participants aged 15 to 54 years.

For this study, of 2 077 704 eligible participants older than 15 years, we excluded participants with missing data or biologically implausible values on each outcome variable. For obesity, we excluded pregnant people at the time of the survey. The final analytic sample included 1 715 895 individuals (921 779 female and 794 116 male) analyzed for hypertension, 1 807 566 (961 977 female and 845 589 male) for diabetes, and 776 023 (678 782 female and 97 241 male) for obesity. eFigure 1 in Supplement 1 describes the exclusion criteria in detail. According to the Harvard Longwood Campus Institutional Review Board, institutional review board review and informed consent were not required for this study because it relied exclusively on publicly accessible secondary use of anonymized information. This cross-sectional study followed the Strengthening the Reporting of Observational Studies in Epidemiology (STROBE) reporting guideline.

### Primary Outcomes

The primary outcomes were hypertension, diabetes, and obesity. Each of these 3 major risk factors of CVD was coded as a binary variable, with 1 indicating the presence of the condition and 0 indicating otherwise.

#### Hypertension

The systolic and diastolic blood pressure was measured 3 times for each participant using a portable blood pressure monitor (HEM-8712, Omron Healthcare Inc).^19^ We discarded the first reading and considered the mean of the last 2 readings to avoid upward bias from anxiety or nervousness while taking the first reading.^20^ Hypertension was defined as having a mean systolic blood pressure of 140 mm Hg or higher or mean diastolic blood pressure of 90 mm Hg or higher^21^ or responding yes to the following question: “Are you currently taking medications prescribed to lower blood pressure?” This question was asked of all participants regardless of their blood pressure measurements.

#### Diabetes

Blood glucose was measured with a portable blood glucose measuring instrument (FreeStyle Optium H, Abbott Laboratories).^22^ Diabetes was defined as having a high plasma-equivalent blood glucose level of 200 mg/dL or higher (to convert to millimoles per liter, multiply by 0.0555)^23^ or reporting yes when asked, “Are you currently taking a prescribed medicine to lower blood glucose?” We adopted a cutoff of 200 mg/dL instead of the general cutoff of 126 mg/dL because random blood glucose measurements were taken, meaning that fasting before the test was not required.^24^

#### Obesity

Height and weight were measured by portable measuring instruments for all participants (Seca 874 digital scale for weight [in kilograms] and Seca 213 stadiometer for height [in centimeters], Seca). Body mass index (BMI) was calculated as weight in kilograms divided by heights in meters squared. For the main analysis, obesity was defined as a BMI of 30 or greater according to World Health Organization classification for the general population.^25^ For sensitivity analysis, we used other BMI cutoffs^26^ (27.5 and 25) considered for Asian populations and alternative anthropometric measures^27^ (waist circumference and waist-to-hip ratio).

### Statistical Analysis

The final analytic sample followed a 4-level hierarchical structure: individual *i* (level 1), small area *j* (level 2), district *k* (level 3), and state or Union Territory *l* (level 4), all of which have political, administrative, and sociocultural significance that may influence population health in India.^5^ In this study, the primary sampling units (equivalent to villages in rural areas and blocks in urban areas) were collectively labeled as small areas.

A 4-level logistic regression was estimated for each outcome using standard multilevel modeling^6,7,8,9,10,11,12,13,14^ (eEquation 1 in Supplement 1), and variance partitioning coefficient (VPC) was calculated for each level (eEquation 2 in Supplement 1). In this study, we focused on the total geographic variability as opposed to total variability (including 3.29 as level 1 variance) to assess the relative importance of geographic levels. For state-specific analysis, a series of 3-level models was computed to estimate the VPCs.

On the basis of the 4-level model above, precision-weighted estimates for each small area were computed (eEquation 3 in Supplement 1). On the basis of these estimates, within-district small area variability was assessed by computing the SD for each district. Similarly, the overall mean prevalence of outcome at the district level was computed (eEquation 4 in Supplement 1). To assess a systematic pattern in district-wide mean prevalence and within-district variability, we visualized them using choropleth maps. Moreover, we calculated the correlation coefficient between the 2 measures in India and across each state. We also classified districts into tertiles—low, medium, and high—based on district-wide mean prevalence and within-district variability to identify the highest priority districts (ie, high prevalence and high SD in CVD risk factors).

In this study, all analyses were stratified by sex and then further stratified by age groups (younger individuals [15-39 years], middle-aged individuals [40-59 years], and older adults [≥60 years]) and place of residence (urban or rural) for supplementary analyses. For all descriptive statistics, survey weights were applied to account for the multistage sampling design. All analyses were conducted using Stata software, version 16 (StataCorp)^28^ and MLwiN, version 3.05 (using runmlwin) (StataCorp).^29^ The configuration files were obtained from the International Institute for Population Sciences, the administrating organization of the NFHS 2019-2021 survey in India. All maps were generated using ArcGIS Pro software, version 3.0 (Esri).^30^

## Results

### Sample Characteristics

The final analytic sample consisted of 1 715 895 individuals analyzed for hypertension (mean [SD] age, 39.8 [17.3] years; 921 779 female [53.7%] and 794 116 male [46.3%]), 1 807 566 for diabetes (mean [SD] age, 39.5 [17.2] years; 961 977 female [53.2%] and 845 589 male [46.8%]), and 776 023 individuals for obesity (mean [SD] age, 30.9 [10.2] years; 678 782 female [87.5%] and 97 241 male [12.5%]). Among women, 21.2% had hypertension, 5.0% had diabetes, and 6.3% had obesity (eTable 1 in Supplement 1). Male participants had a slightly higher prevalence of hypertension (24.1%) and diabetes (5.4%) but a lower prevalence of obesity (4.0%) compared with women. The prevalence of CVD risk factors was higher for older individuals; for example, the prevalence of hypertension among female participants was 7.6% in younger individuals, 30.7% in middle-aged individuals, and 51.3% in older individuals. The overall distribution of outcomes was greater for small areas compared with districts or states (eTable 2 in Supplement 1). The IQR of hypertension prevalence among women was largest across small areas (13.8%-28.6% [median, 20.7%]), followed by districts (18.0%-24.9% [median, 21.4%]), and states (20.1%-24.5% [median, 22.3%]). The same was true for diabetes and obesity in both men and women. For brevity, results are presented for female participants from hereon, and findings for male participants are noted only when sex inconsistency is found.

### Hypertension

**Figure 1.**
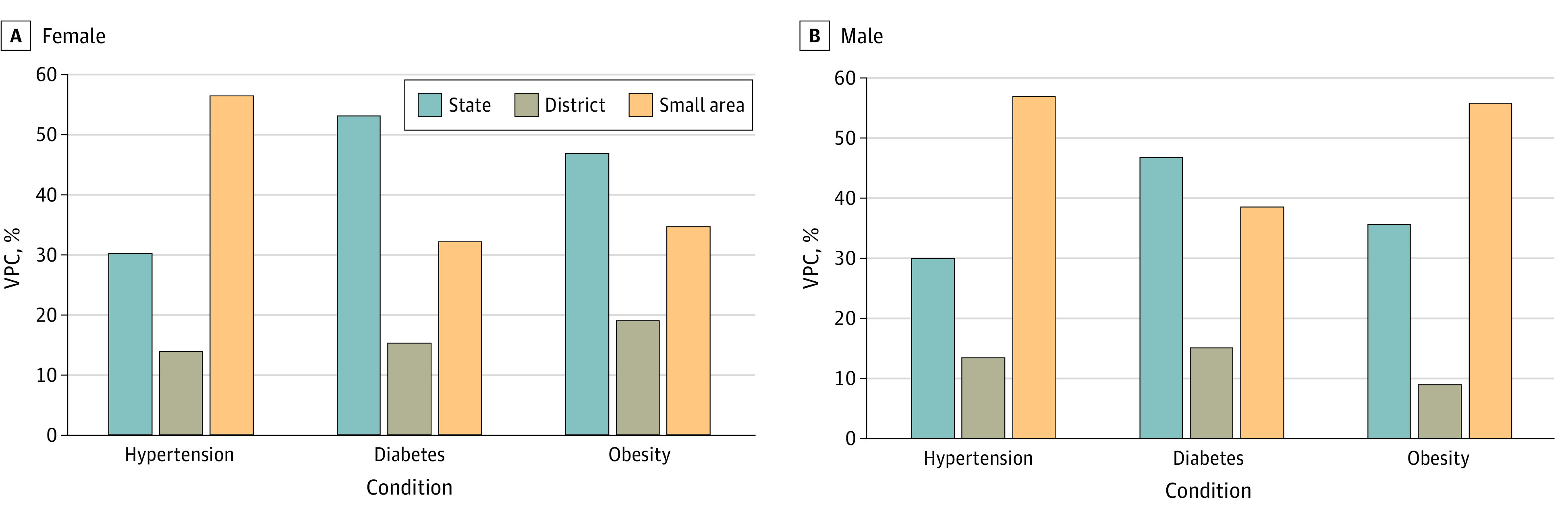
Geographic Variance Partitioning for Cardiovascular Disease Risk Factors, Stratified by Sex VPC indicates variance partitioning coefficient.

**Figure 2.**
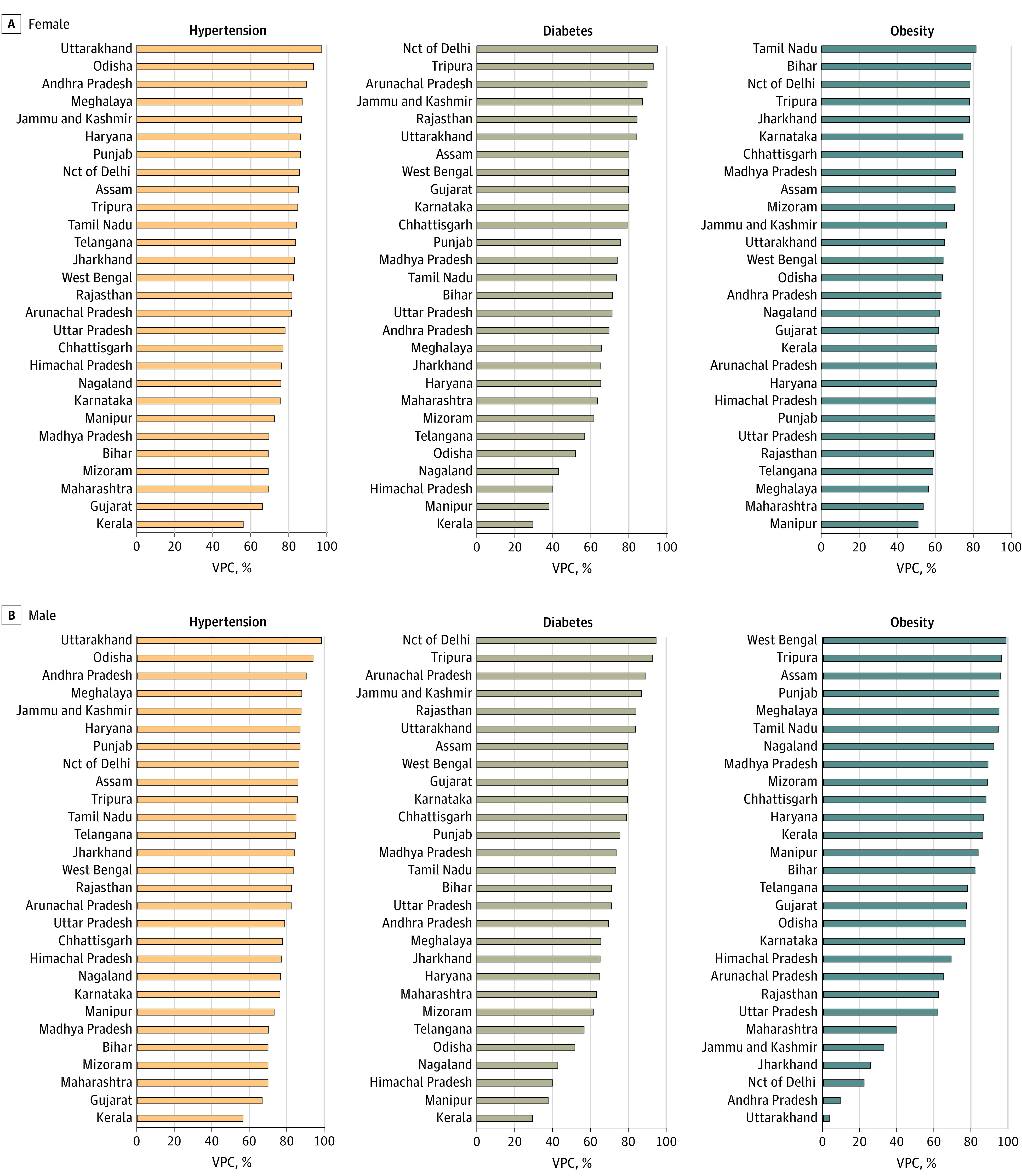
Geographic Variability Attributable to Small Areas Across States, Stratified by Sex Due to the small sample size, 8 states with fewer than 5 districts (Chandigarh, Sikkim, Dadra and Nagar Haveli and Daman and Diu, Goa, Lakshadweep, Puducherry, Andaman and Nicobar Islands, and Ladakh) were excluded from the state-specific analysis. Nct indicates National Capital Territory; VPC, variance partitioning coefficient.

A total of 56.2% of the total geographic variability in hypertension was attributed to small areas, followed by 30.0% to states and 13.8% to districts (Figure 1; eTable 3 in Supplement 1). In addition, small area variability was substantial across all states (Figure 2). For example, more than 50% of the total geographic variability was attributed to small areas for all states and more than 80% in half of the states.

**Figure 3.**
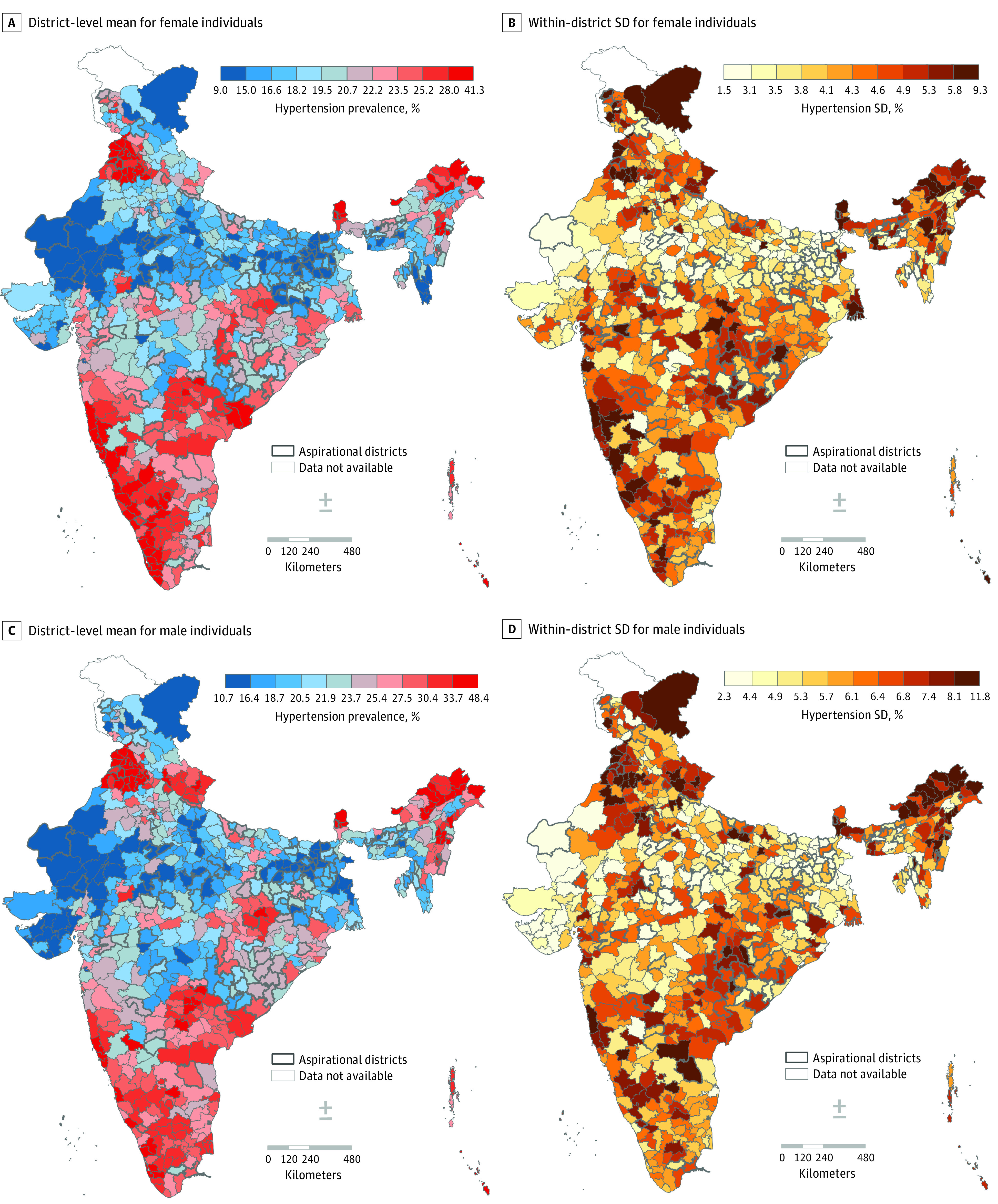
District-Wide Mean and Within-District Variability in Hypertension, Stratified by Sex

The correlation between district-wide mean and within-district small area variability was moderate at best (*r* = 0.66) (eFigure 2 in Supplement 1). The correlation coefficient varied widely across states, ranging from 0.03 to 0.85 (eTable 4 in Supplement 1). Among male participants, the range of correlation coefficients across states was wider, as there was even a negative correlation in Kerala (*r* = −0.22) and Chhattisgarh (*r* = −0.17) (eTable 5 in Supplement 1). Overall, districts in western India appear to have lower means for hypertension but substantial within-district variability (Figure 3). On the basis of district-wide mean and within-district variability, 150 districts were identified as highest-priority areas, whereas 286 districts had discordance in the level of overall prevalence and small area variability (eTable 6 in Supplement 1).

### Diabetes

**Figure 4.**
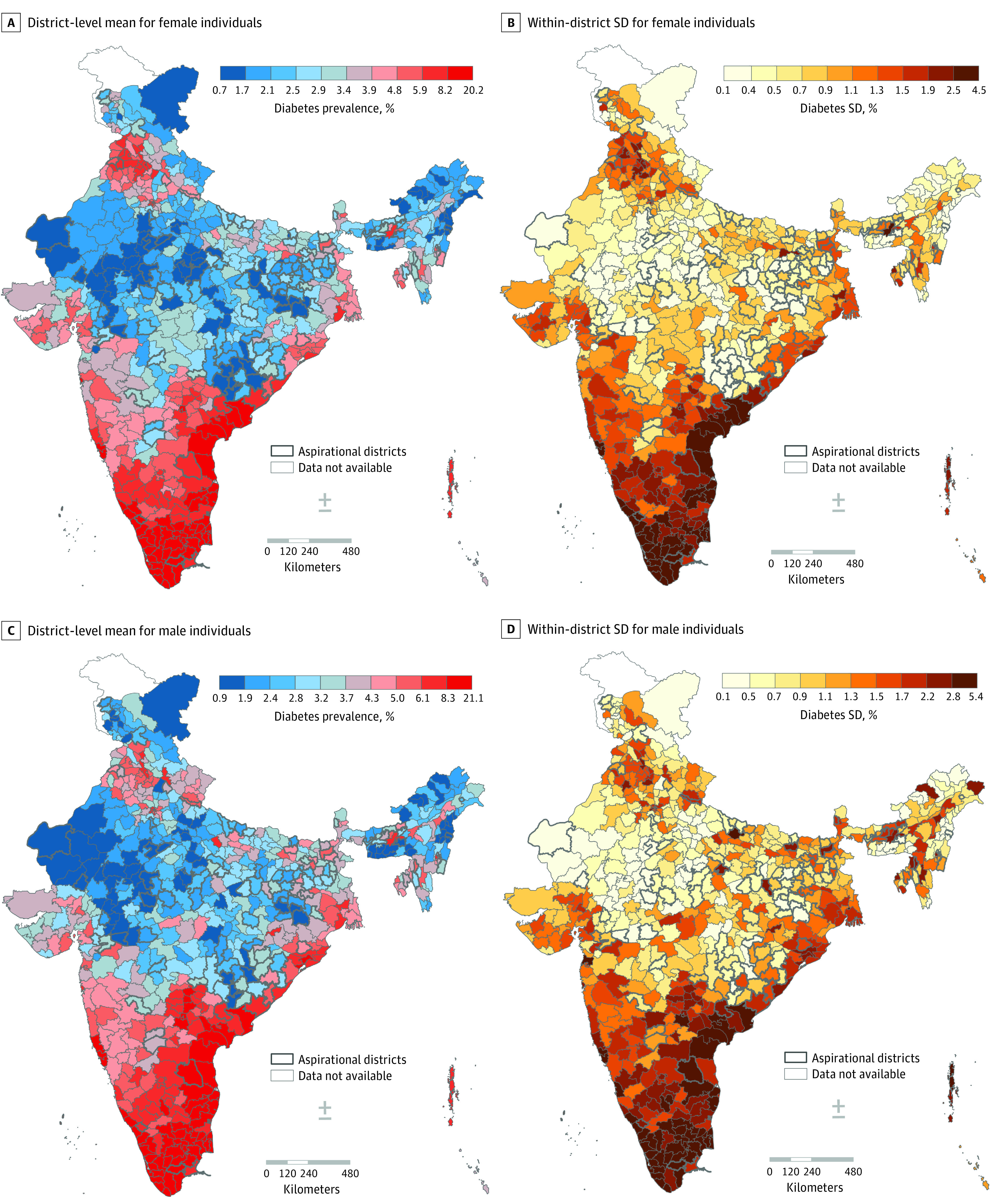
District-Wide Mean and Within-District Variability in Diabetes, Stratified by Sex

States contributed the most to the total geographic variability in diabetes (52.8%), followed by small areas (32.0%), and districts (15.2%) (Figure 1). According to state-specific analysis, in 25 of 28 states, small areas accounted for more than half of the geographic variability (Figure 2). A strong positive correlation was found between district-wide mean and within-district small area variability (*r* = 0.96) (eFigure 3 in Supplement 1). In state-specific analysis, correlations were consistently substantial, as 23 states had correlations greater than 0.8 (eTable 4 in Supplement 1). When visualized in maps, highest-priority districts with high mean prevalence and high within-district variability were considerably overlapping (Figure 4). For instance, the highest-priority districts were largely concentrated in the southern region, including Kerala, Tamil Nadu, and Andhra Pradesh. Furthermore, there was a greater concordance between high mean and high SD for diabetes compared with hypertension, as 589 of 707 districts were classified in the same tertile (eTable 6 in Supplement 1).

### Obesity

**Figure 5.**
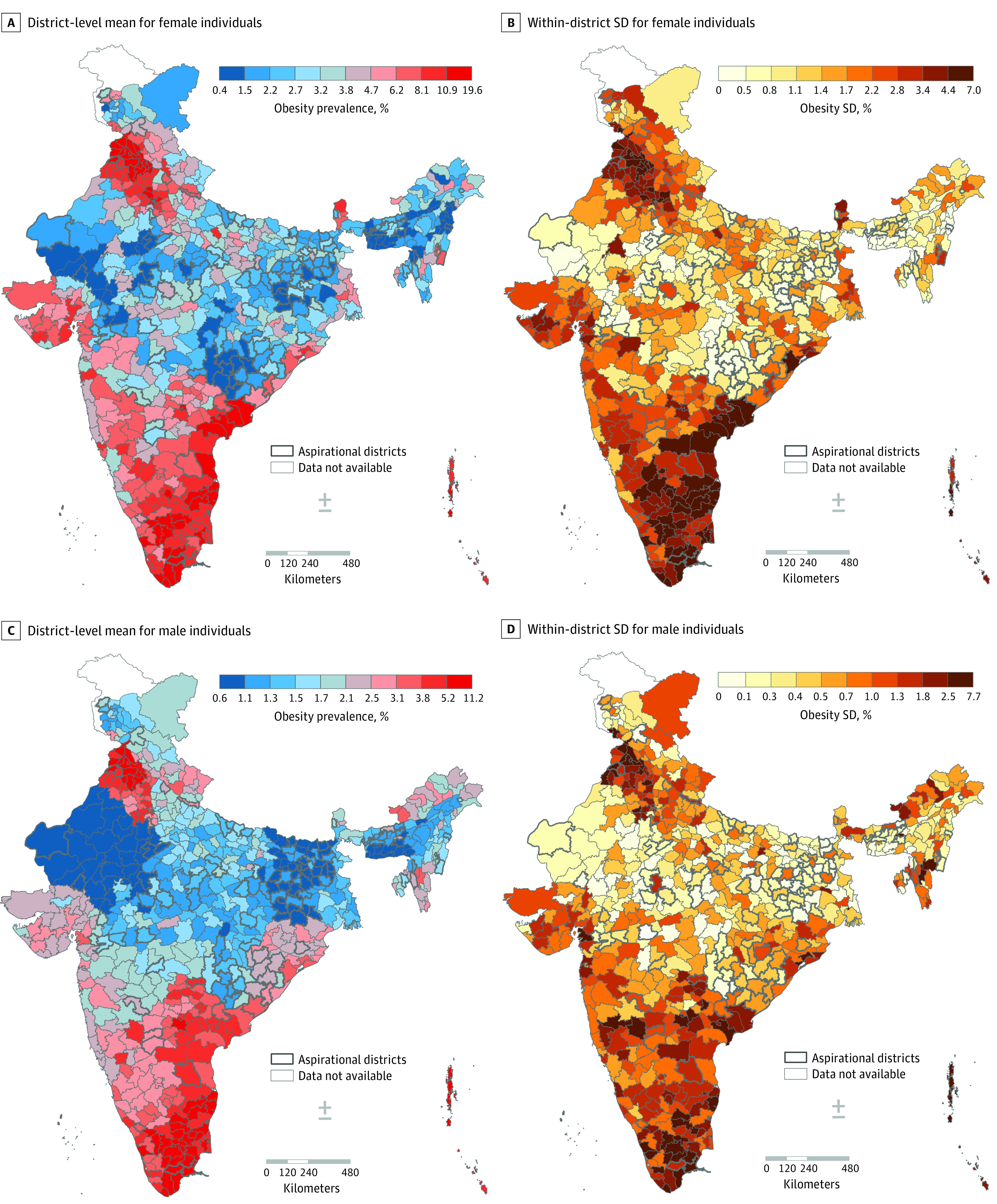
District-Wide Mean and Within-District Variability in Obesity, Stratified by Sex

Small areas (34.5%) contributed greatly to the total geographic variability in obesity, second only to states (46.6%) but much greater than districts (18.9%) (Figure 1). For male participants, the proportion of small area variability was the largest (55.6%), followed by states (35.5%) and then districts (8.9%). For most of the states, small areas accounted for more than 50% of the geographic variability for both sexes (Figure 2). When using other BMI cutoffs, waist circumference, and waist-to-hip ratio, small areas and states continued to be the largest source of geographic variability (eTable 7 in Supplement 1). Strong positive correlation was observed between district-wide means and small area variability (*r* = 0.94), as well as strong correlation across each state, with 23 of 28 states having correlations greater than 0.8. (eFigure 4; eTable 4 in Supplement 1). Substantial concordance between high mean and high SD was observed (605 of 707 districts), and districts with high mean prevalence and high SD were concentrated in the southern region (eTable 6 in Supplement 1; Figure 5).

### Supplementary Analysis

In the age-stratified analysis, a consistent pattern was found for all outcomes. The proportion of small area and state variability was most substantial across all age groups, except for diabetes among younger participants and obesity among middle-aged men (eTables 8-10 in Supplement 1). In addition, across all age groups, correlations between district-wide mean and within-district small area variability were comparable to the main findings except for hypertension (eFigures 5-12 in Supplement 1). Notably, for hypertension, older adults participants (*r* = 0.11) had a weaker correlation than middle-aged participants (*r* = 0.61) and younger participants (*r* = 0.86) (eFigures 5-7 in Supplement 1). Similarly, in hypertension, greater discordance was found in district-wide means and SDs among older participants (498 of 707 districts) (eTables 11-13 in Supplement 1). Regarding place of residence, the VPC result was comparable to the main findings for both urban and rural areas (eTable 14 in Supplement 1).

## Discussion

To our knowledge, this is the first study to examine and quantify the geographic variability of CVD risk factors at 3 key geographic levels in India: state, district, and small area. Our findings indicate that states and small areas contributed the most to geographic variability, whereas districts contributed the least for all CVD risk factors. In addition, strong positive correlations between district-wide mean and within-district variability suggested that districts with a high overall burden of CVD risk factors were also likely to have greater inequalities. These findings underscore that small areas should be considered when designing policies and programs to prevent and manage CVD risk factors in India.

The relative importance of state variability has been previously demonstrated for NCDs, including cancer and CVD risk factors.^17,31,32,33,34^ The substantial heterogeneity across states is expected because almost all states in India have a population size larger than most other countries, with Uttar Pradesh being larger than Brazil.^35^ In addition, varying degrees of economic and social development, environments, and population structure across states can ultimately result in considerable state heterogeneity.^32^ This supports the importance of considering state-specific strategies for efficient implementation of health programs. In addition to well-established evidence on the importance of state-level data, our findings underscore considering substantial small area variability within districts. The large VPC in small areas was consistent across most states for both sexes, all age groups, and urban vs rural areas, indicating strong contextual effects of local environments on CVD risk factors. This finding emphasizes the relative importance of smaller geographic units in implementing and intervening in CVD risk factor prevention programs.

Current policies and interventions in India are primarily based on the district level.^4,5^ For instance, approximately 13 direct and indirect national programs are related to preventing and controlling NCDs, all based on states and districts.^36^ The National Programme for Prevention and Control of Cancer, Diabetes, Cardiovascular Diseases and Stroke, one of India’s main national programs for NCD, was initiated in 2010 with a pilot phase that included 10 districts and gradually expanded to cover all the districts,^36^ with management units being established at the district level.^4^ Despite these efforts, the performance and efficacy of these programs were relatively poor.^36^ Our findings on the small contribution of districts in geographic variability in CVD risk factors indicate that more prevention and control programs that explicitly take into account the smaller geographic areas are required. Ideally, policy design and initiation based on macro units combined with adaptation, implementation, and monitoring systems based on micro units could optimize the prevention and treatment of CVD risk factors.

Our findings of strong positive correlations between district-wide mean and within-district small area variability indicate that districts with higher means also tend to have greater health inequalities in CVD risk factors. At the same time, for hypertension, moderate correlation and discordance in the district classification suggest that the current approach in identifying highest-priority districts solely based on district-wide prevalence may overlook districts with above-average performance but significant health inequalities. Interestingly, for hypertension among older adults, there were almost null correlations between district-wide mean and within-district variability and greater discordance in the district classification. In this case, districts with low district-wide mean prevalence but large within-district inequalities should be prioritized in policy formulation for efficiency. Prioritizing the most vulnerable small areas would not only contribute to the overall progress toward reducing the burden of CVD in India but also reduce inequality within districts, thereby ensuring that India upholds the principle of universality as committed by the Sustainable Development Goals.^37^

### Limitations

This study has several limitations. First, although rural villages and urban blocks cannot be directly compared because of different sizes, administrative roles, and other features,^38^ we treated them as a collective unit of small areas for the purpose of our analysis in quantifying the importance of multiple micro and macro units in India. Although our sensitivity analysis produced consistent results, future investigations should consider the differential growth of some of the CVD risk factors by urban and rural areas. Second, all estimates in this study were not adjusted for demographic and socioeconomic covariates associated with CVD risk factors.^39^ This was a deliberate choice, allowing us to focus on the crude geographic distribution of CVD risk factors to assess the baseline contextual importance. Adjusting for age, educational level, wealth, and place of residence did not change the study’s findings. More in-depth examination of the association among demographic, socioeconomic covariates, and CVD risk factors is beyond the scope of this article and should be considered in future studies. Third, the use of nonfasting samples might not accurately capture the prevalence of diabetes,^40^ especially in India, with varying meal timings and dietary habits across a large geographic area. However, random blood glucose testing is often the more affordable and realistic option for a large-scale survey in low- and middle-income countries.^24^ Fourth, it is plausible that the substantial small area variability we found is, to some extent, an artifact of a few areas with extreme values. Although multilevel modeling has technical advantages in small area estimation by leveraging information from other areas within the same district,^41^ it does not completely rule out the influence of outliers. Thus, we emphasize that small area variability should always be interpreted together with the overall mean of the district when determining the priority areas for resource allocation.

## Conclusions

This cross-sectional study underscores substantial small area variability within districts and the already well-acknowledged macro-level variability among states in India. Future research should be directed toward identifying and targeting specific small areas and their distinct characteristics associated with CVD risk factors, enabling the development of more tailored interventions. With a decrease in the age at onset of CVD risk factors, as evidenced by decreasing median ages for hypertension and diabetes in India,^42^ addressing the burden of NCD-related morbidity and premature mortality becomes imperative because of their associated economic and societal implications.^43,44^ To effectively design and precisely implement interventions for CVD risk factors, it is necessary to focus on micro units, considering both the mean performance of districts and small area variability.

## Notes

Supplement 1. **eEquation 1.** Four-level logistic regression **eEquation 2.** Variance partitioning coefficient **eEquation 3.** Precision-weighted estimates for small area variability **eEquation 4.** Precision-weighted estimates for district-wide mean **eTable 1.** Descriptive statistics of study participants **eTable 2.** Distribution of CVD risk factors, stratified by sex **eTable 3.** Geographic variance partitioning for CVD risk factors along with variance and standard errors, stratified by sex **eTable 4.** State-specific correlation between district-wide mean and within-district variability of CVD risk factors among women **eTable 5.** State-specific correlation between district-wide mean and within-district variability of CVD risk factors among men **eTable 6.** Distribution of districts based on the district-wide mean and within district variability of CVD risk factors, stratified by sex **eTable 7.** Geographic variance partitioning using different standards of obesity, stratified by sex **eTable 8.** Geographic variance partitioning for CVD risk factors along with variance and standard errors among younger individuals, stratified by sex **eTable 9.** Geographic variance partitioning for CVD risk factors along with variance and standard errors among middle-aged individuals, stratified by sex **eTable 10.** Geographic variance partitioning for CVD risk factors along with variance and standard errors among older adults, stratified by sex **eTable 11.** Distribution of districts based on the district-wide mean and within district variability of CVD risk factors among younger individuals, stratified by sex **eTable 12.** Distribution of districts based on the district-wide mean and within district variability of CVD risk factors among middle-aged individuals, stratified by sex **eTable 13.** Distribution of districts based on the district-wide mean and within district variability of CVD risk factors among older adults, stratified by sex **eTable 14.** Geographic variance partitioning, stratified by sex and place of residence **eFigure 1.** Flow diagram showing exclusions and final sample sizes for primary analysis of the study population **eFigure 2.** Correlation between district-wide mean and within-district variability in hypertension, stratified by sex **eFigure 3.** Correlation between district-wide mean and within-district variability in diabetes, stratified by sex **eFigure 4.** Correlation between district-wide mean and within-district variability in obesity, stratified by sex **eFigure 5.** District-wide mean, within-district variability, and correlation between them in hypertension among younger individuals, stratified by sex **eFigure 6.** District-wide mean, within-district variability, and correlation between them in hypertension among middle-aged individuals, stratified by sex **eFigure 7.** District-wide mean, within-district variability, and correlation between them in hypertension among older adults, stratified by sex **eFigure 8.** District-wide mean, within-district variability, and correlation between them in diabetes among younger individuals, stratified by sex **eFigure 9.** District-wide mean, within-district variability, and correlation between them in diabetes among middle-aged individuals, stratified by sex. **eFigure 10.** District-wide mean, within-district variability, and correlation between them in diabetes among older adults, stratified by sex. **eFigure 11.** District-wide mean, within-district variability, and correlation between them in obesity among young individuals, stratified by sex. **eFigure 12.** District-wide mean, within-district variability, and correlation between them in obesity among middle-aged individuals, stratified by sex.

Supplement 2.
Data Sharing Statement
