# Supplementary material for: Small Area Geographic Estimates of Cardiovascular Disease Risk Factors in India
Source: JAMA Netw Open. 2023 Oct 12;6(10):e2337171. doi: 10.1001/jamanetworkopen.2023.37171 (PMC10570875; doi:10.1001/jamanetworkopen.2023.37171)
Supplement: Supplement 1. — eEquation 1. Four-level logistic regression eEquation 2. Variance partitioning coefficient eEquation 3. Precision-weighted estimates for small area variability eEquation 4. Precision-weighted estimates for district-wide mean eTable 1. Descriptive statistics of study participants eTable 2. Distribution of CVD risk factors, stratified by sex eTable 3. Geographic variance partitioning for CVD risk factors along with variance and standard errors, stratified by sex eTable 4. State-specific correlation between district-wide mean and within-district variability of CVD risk factors among women eTable 5. State-specific correlation between district-wide mean and within-district variability of CVD risk factors among men eTable 6. Distribution of districts based on the district-wide mean and within district variability of CVD risk factors, stratified by sex eTable 7. Geographic variance partitioning using different standards of obesity, stratified by sex eTable 8. Geographic variance partitioning for CVD risk factors along with variance and standard errors among younger individuals, stratified by sex eTable 9. Geographic variance partitioning for CVD risk factors along with variance and standard errors among middle-aged individuals, stratified by sex eTable 10. Geographic variance partitioning for CVD risk factors along with variance and standard errors among older adults, stratified by sex eTable 11. Distribution of districts based on the district-wide mean and within district variability of CVD risk factors among younger individuals, stratified by sex eTable 12. Distribution of districts based on the district-wide mean and within district variability of CVD risk factors among middle-aged individuals, stratified by sex eTable 13. Distribution of districts based on the district-wide mean and within district variability of CVD risk factors among older adults, stratified by sex eTable 14. Geographic variance partitioning, stratified by sex and place of residence eFigure 1. Flow d [file jamanetwopen-e2337171-s001.pdf]

Supplemental Online Content

Ko S, Oh H, Subramanian SV, Kim R. Small area geographic estimates of cardiovascular disease risk factors in India. *JAMA Netw Open*. 2023;6(10):e2337171. doi:10.1001/jamanetworkopen.2023.37171

eEquation 1. Four-level logistic regression.
eEquation 2. Variance partitioning coefficient.
eEquation 3. Precision-weighted estimates for small area variability.
eEquation 4. Precision-weighted estimates for district-wide mean.
eTable 1. Descriptive statistics of study participants.
eTable 2. Distribution of CVD risk factors, stratified by sex.
eTable 3. Geographic variance partitioning for CVD risk factors along with variance and standard errors, stratified by sex.
eTable 4. State-specific correlation between district-wide mean and within-district variability of CVD risk factors among women.
eTable 5. State-specific correlation between district-wide mean and within-district variability of CVD risk factors among men.
eTable 6. Distribution of districts based on the district-wide mean and within district variability of CVD risk factors, stratified by sex.
eTable 7. Geographic variance partitioning using different standards of obesity, stratified by sex.
eTable 8. Geographic variance partitioning for CVD risk factors along with variance and standard errors among younger individuals, stratified by sex.
eTable 9. Geographic variance partitioning for CVD risk factors along with variance and standard errors among middle-aged individuals, stratified by sex.
eTable 10. Geographic variance partitioning for CVD risk factors along with variance and standard errors among older adults, stratified by sex.
eTable 11. Distribution of districts based on the district-wide mean and within district variability of CVD risk factors among younger individuals, stratified by sex.
eTable 12. Distribution of districts based on the district-wide mean and within district variability of CVD risk factors among middle-aged individuals, stratified by sex.
eTable 13. Distribution of districts based on the district-wide mean and within district variability of CVD risk factors among older adults, stratified by sex.
eTable 14. Geographic variance partitioning, stratified by sex and place of residence.
eFigure 1. Flow diagram showing exclusions and final sample sizes for primary analysis of the study population.
eFigure 2. Correlation between district-wide mean and within-district variability in hypertension, stratified by sex.
eFigure 3. Correlation between district-wide mean and within-district variability in diabetes, stratified by sex.
eFigure 4. Correlation between district-wide mean and within-district variability in obesity, stratified by sex.
eFigure 5. District-wide mean, within-district variability, and correlation between them in hypertension among younger individuals, stratified by sex.
eFigure 6. District-wide mean, within-district variability, and correlation between them in hypertension among middle-aged individuals, stratified by sex.
eFigure 7. District-wide mean, within-district variability, and correlation between them in hypertension among older adults, stratified by sex.
eFigure 8. District-wide mean, within-district variability, and correlation between them in diabetes among younger individuals, stratified by sex.

© 2023 Ko S et al. *JAMA Network Open.*

eFigure 9. District-wide mean, within-district variability, and correlation between them in diabetes among middle-aged individuals, stratified by sex.
eFigure 10. District-wide mean, within-district variability, and correlation between them in diabetes among older adults, stratified by sex.
eFigure 11. District-wide mean, within-district variability, and correlation between them in obesity among young individuals, stratified by sex.
eFigure 12. District-wide mean, within-district variability, and correlation between them in obesity among middle-aged individuals, stratified by sex.

This supplementary material has been provided by the authors to give readers additional information about their work

© 2023 Ko S et al. *JAMA Network Open.*

eEquation 1. Four-level logistic regression.

A four-level logistic regression was estimated for each of the CVD risk factors (Y_{ijkl}):

$$\mathrm{L}og(\mathrm{P}(\mathrm{Y}_{ijkl}{=}1))/(1\text{-}\ \mathrm{P}(\mathrm{Y}_{ijkl}{=}1))\ =\ \beta_0+u_{0jkl}+v_{0kl}+f_{0l}$$
$$u_{0jkl}\sim\mathrm{N}(0,\ \sigma^2_{u0})$$
$$v_{0kl}\sim\mathrm{N}(0,\ \sigma^2_{v0})$$
$$f_{0l}\sim\mathrm{N}(0,\ \sigma^2_{f0})$$

In this model, u_{0jkl}, v_{0kl}, and f_{0l} are residuals for small are, district, and state, respectively. The term σ^2_{u0} denotes within-district, between-small area variance; σ^2_{v0} represents within-state, between-district variance; and σ^2_{f0} is the between-state variance. For binary outcomes, individual-level variance is not directly estimated and instead assumed to come from a logistic distribution with a fixed variance of $\pi^2/3$ or 3.29. Multilevel analysis was performed via Monte Carlo Markov Chain (MCMC) methods using Gibbs sampler with the default prior distributions of Iterated Generalized Least Squares (IGLS) estimations as starting values, a burn-in of 500 cycles, and monitoring of 5000 iterations of chains.

© 2023 Ko S et al. *JAMA Network Open.*

eEquation 2. Variance partitioning coefficient.

Variance partitioning coefficient (VPC) was calculated for each level *z* as:

$$\left(\frac{\sigma_z^2}{\sigma_{u0}^2 + \sigma_{v0}^2 + \sigma_{f0}^2}\right) \times 100$$

© 2023 Ko S et al. *JAMA Network Open.*

eEquation 3. Precision-weighted estimates for small area variability.

Precision-weighted estimates of outcome Y for each small area were computed as:

$$\exp(\beta_0 + u_{0jkl} + v_{0kl} + f_{0l})/(1+\exp(\beta_0 + u_{0jkl} + v_{0kl} + f_{0l}))$$

© 2023 Ko S et al. *JAMA Network Open.*

eEquation 4. Precision-weighted estimates for district-wide mean.

The overall mean probability of outcome Y at the district level was computed, excluding small area-specific residuals (u_{0jkl}):

$$\exp(\beta_0 + v_{0kl} + f_{0l})/(1+\exp(\beta_0 + v_{0kl} + f_{0l}))$$

© 2023 Ko S et al. *JAMA Network Open.*

eTable 1. Descriptive statistics of study participants.

Outcome	Distribution	Frequency	Prevalence (%)
**Hypertension**			
Total Women	921,779	195,087	21.22
Women (15-39 years)	507,848	41,287	7.64
Women (40-59 years)	275,852	84,396	30.68
Women (60-95 years)	138,079	69,404	51.29
**Diabetes**			
Total Women	961,977	39,732	4.96
Women (15-39 years)	539,417	6,050	1.27
Women (40-59 years)	284,597	18,414	7.86
Women (60-95 years)	137,963	15,268	12.98
**Obesity**			
Total Women	678,782	36,599	6.31
Women (15-39 years)	517,346	21,600	4.90
Women (40-49 years)	161,436	14,999	10.77
**Hypertension**			
Total Men	794,116	194,921	24.12
Men (15-39 years)	418,117	53,802	12.32
Men (40-59 years)	237,870	78,376	32.21
Men (60-95 years)	138,129	62,743	45.48
**Diabetes**			
Total Men	845,589	38,291	5.36
Men (15-39 years)	454,481	6,004	1.49
Men (40-59 years)	249,847	16,710	7.96
Men (60-95 years)	141,261	15,577	12.98
**Obesity**			
Total Men	97,241	3,562	4.00
Men (15-39 years)	68,158	2,034	3.37
Men (40-54 years)	29,083	1,528	5.50

Note. To account for the multistage sampling design, survey weights were applied to all descriptive statistics.

© 2023 Ko S et al. *JAMA Network Open.*

eTable 2. Distribution of CVD risk factors, stratified by sex.

	Min	5th	25th	50th	75th	95th	Max
**Women**							
**Hypertension**							
Small area	0.0	5.3	13.8	20.7	28.6	43.3	100.0
District	8.5	14.1	18.0	21.4	24.9	30.4	42.9
State	15.0	17.1	20.1	22.3	24.5	30.5	37.3
**Diabetes**							
Small area	0.0	0.0	0.0	3.4	7.9	17.6	75.0
District	0.4	1.6	2.8	4.2	6.6	12.3	21.9
State	1.6	2.4	3.9	4.0	6.9	14.2	14.2
**Obesity**							
Small area	0.0	0.0	0.0	4.3	10.3	25.0	100.0
District	0.1	1.6	3.3	5.2	9.4	16.0	21.7
State	1.3	2.6	4.6	4.7	9.8	14.3	16.9
**Men**							
**Hypertension**							
Small area	0.0	4.9	15.4	24.0	33.3	50.0	100.0
District	10.1	15.2	19.8	24.3	28.9	35.2	48.9
State	17.4	19.9	21.5	23.0	30.6	32.8	45.8
**Diabetes**							
Small area	0.0	0.0	0.0	4.0	8.7	20.0	100.0
District	0.3	1.8	3.3	4.7	7.1	12.9	22.9
State	1.8	2.9	4.3	5.0	6.8	15.2	15.2
**Obesity**							
Small area	0.0	0.0	0.0	0.0	7.7	22.2	100.0
District	0.0	0.4	1.8	3.5	6.2	11.5	21.7
State	1.0	1.9	2.9	3.7	6.1	9.0	11.2

Note. To account for the multistage sampling design, survey weights were applied to all descriptive statistics.

© 2023 Ko S et al. *JAMA Network Open.*

eTable 3. Geographic variance partitioning for CVD risk factors along with variance and standard errors, stratified by sex.

	Hypertension		Diabetes		Obesity	
	Var (SE)	VPC (%)	Var (SE)	VPC (%)	Var (SE)	VPC (%)
**Women**						
State	0.085 (0.023)	30	0.403 (0.109)	52.8	0.569 (0.152)	46.6
District	0.039 (0.003)	13.8	0.116 (0.008)	15.2	0.231 (0.016)	18.9
Small area[1]	0.159 (0.003)	56.2	0.244 (0.008)	32	0.422 (0.012)	34.5
**Men**						
State	0.120 (0.034)	29.9	0.367 (0.097)	46.6	0.392 (0.111)	35.5
District	0.054 (0.004)	13.4	0.118 (0.009)	15	0.094 (0.020)	8.9
Small area[2]	0.227 (0.004)	56.7	0.302 (0.009)	38.4	0.617 (0.055)	55.6

Note. Var=variance; SE=standard error; VPC=variance partitioning coefficient
[1]: Hypertension: N=29,920; Diabetes: N= 30,122; Obesity: N=30,117
[2]: Hypertension: N=29,869; Diabetes: N= 30,114; Obesity: N=9,074

© 2023 Ko S et al. *JAMA Network Open.*

eTable 4. State-specific correlation between district-wide mean and within-district variability of CVD risk factors among women.

State	No. of Districts	Hypertension	Diabetes	Obesity
Jammu & Kashmir	20	0.28	0.73	0.88
Himachal Pradesh	12	0.82	0.95	0.85
Punjab	22	0.24	0.87	0.79
Chandigarh	1			
Uttarakhand	13	0.70	0.92	0.67
Haryana	22	0.72	0.89	0.89
Nct Of Delhi	11	0.74	0.44	0.91
Rajasthan	33	0.58	0.86	0.92
Uttar Pradesh	75	0.64	0.91	0.92
Bihar	38	0.76	0.93	0.86
Sikkim	4			
Arunachal Pradesh	20	0.65	0.78	0.92
Nagaland	11	0.61	0.89	0.74
Manipur	9	0.35	0.96	0.92
Mizoram	8	0.81	0.99	0.69
Tripura	8	0.85	0.87	0.99
Meghalaya	11	0.03	0.97	0.94
Assam	33	0.67	0.89	0.95
West Bengal	20	0.70	0.84	0.95
Jharkhand	24	0.33	0.92	0.85
Odisha	30	0.41	0.89	0.82
Chhattisgarh	27	0.24	0.89	0.91
Madhya Pradesh	51	0.71	0.81	0.85
Gujarat	33	0.74	0.9	0.86
Dadra & Nagar Haveli And Daman & Diu	3			
Maharashtra	36	0.63	0.9	0.88
Andhra Pradesh	13	0.50	0.92	0.93
Karnataka	30	0.78	0.95	0.83
Goa	2			
Lakshadweep	1			
Kerala	14	0.43	0.9	0.87
Tamil Nadu	32	0.67	0.74	0.72
Puducherry	4			
Andaman & Nicobar Islands	3			
Telangana	31	0.29	0.89	0.92
Ladakh	2			

Note. Due to the small sample size, 8 states with fewer than 5 districts (Chandigarh, Sikkim, Dadra and Nagar Haveli and Daman and Diu, Goa, Lakshadweep, Puducherry, Andaman and Nicobar Islands, and Ladakh) were excluded from the state-specific analysis.

© 2023 Ko S et al. *JAMA Network Open.*

eTable 5. State-specific correlation between district-wide mean and within-district variability of CVD risk factors among men.

State	No. of Districts	Hypertension	Diabetes	Obesity
Jammu & Kashmir	20	0.39	0.68	0.97
Himachal Pradesh	12	0.27	0.97	0.88
Punjab	22	0.53	0.64	0.62
Chandigarh	1			
Uttarakhand	13	0.50	0.86	0.82
Haryana	22	0.45	0.83	0.92
Nct Of Delhi	11	0.49	0.69	0.74
Rajasthan	33	0.70	0.82	0.74
Uttar Pradesh	75	0.61	0.77	0.93
Bihar	38	0.77	0.88	0.86
Sikkim	4			
Arunachal Pradesh	20	0.43	0.48	0.83
Nagaland	11	0.53	0.98	0.94
Manipur	9	0.73	0.98	0.84
Mizoram	8	0.40	0.97	0.74
Tripura	8	0.92	0.84	0.85
Meghalaya	11	0.66	0.95	0.96
Assam	33	0.62	0.8	0.78
West Bengal	20	0.75	0.8	0.69
Jharkhand	24	0.36	0.83	0.82
Odisha	30	0.32	0.93	0.88
Chhattisgarh	27	-0.17	0.86	0.76
Madhya Pradesh	51	0.62	0.9	0.77
Gujarat	33	0.71	0.83	0.90
Dadra & Nagar Haveli And Daman & Diu	3			
Maharashtra	36	0.70	0.87	0.92
Andhra Pradesh	13	0.70	0.94	0.90
Karnataka	30	0.69	0.86	0.73
Goa	2			
Lakshadweep	1			
Kerala	14	-0.22	0.85	0.84
Tamil Nadu	32	0.22	0.82	0.74
Puducherry	4			
Andaman & Nicobar Islands	3			
Telangana	31	0.33	0.91	0.76
Ladakh	2			

Note. Due to the small sample size, 8 states with fewer than 5 districts (Chandigarh, Sikkim, Dadra and Nagar Haveli and Daman and Diu, Goa, Lakshadweep, Puducherry, Andaman and Nicobar Islands, and Ladakh) were excluded from the state-specific analysis.

© 2023 Ko S et al. *JAMA Network Open.*

eTable 6. Distribution of districts based on the district-wide mean and within district variability of CVD risk factors, stratified by sex.

	Standard Deviation		
Mean	Low	Medium	High
**Women**			
**Hypertension**			
Low	163	55	18
Medium	61	108	67
High	12	73	150
**Diabetes**			
Low	206	30	0
Medium	30	177	29
High	0	29	206
**Obesity**			
Low	210	26	0
Medium	26	185	25
High	0	25	210
**Men**			
**Hypertension**			
Low	158	53	25
Medium	65	105	66
High	13	78	144
**Diabetes**			
Low	206	27	3
Medium	30	174	32
High	0	35	200
**Obesity**			
Low	198	37	0
Medium	38	168	30
High	0	30	205

© 2023 Ko S et al. *JAMA Network Open.*

eTable 7. Geographic variance partitioning using different standards of obesity, stratified by sex.

	VPC (%)				
	BMI≥30[1]	BMI≥27.5	BMI≥25	WC>88[1]	WHR≥0.85[1]
**Women**					
State	46.6	45.9	43.6	40.3	26.1
District	18.9	18.2	17.6	14.9	11.6
Small area	34.5	35.9	38.8	44.8	62.3
**Men**	BMI≥30[1]	BMI≥27.5	BMI≥25	WC>102[1]	BMI≥0.90[1]
State	35.5	36.5	38.1	40.7	24.7
District	8.9	10.6	12	9.5	8.7
Small area	55.6	52.9	50	49.8	66.6

Note. VPC=variance partitioning coefficient
[1]: Definition for the general population by World Health Organization

© 2023 Ko S et al. *JAMA Network Open.*

eTable 8. Geographic variance partitioning for CVD risk factors along with variance and standard errors among younger individuals, stratified by sex.

	Hypertension		Diabetes		Obesity	
	Var (SE)	VPC (%)	Var (SE)	VPC (%)	Var (SE)	VPC (%)
**Women**						
State	0.083 (0.024)	19.4	0.147 (0.044)	57.7	0.583 (0.162)	49.2
District	0.060 (0.005)	14	0.108 (0.013)	42.2	0.215 (0.016)	18.1
Small area[1]	0.285 (0.011)	66.6	0.0002 (0.00005)	0.1	0.387 (0.014)	32.6
**Men**						
State	0.125 (0.036)	60.7	0.162 (0.047)	55.7	0.399 (0.118)	33.2
District	0.072 (0.005)	14.4	0.129 (0.014)	44.2	0.128 (0.031)	10.6
Small area[2]	0.304 (0.009)	25	0.0001 (0.00003)	0	0.676 (0.077)	56.2

Note. Var=variance; SE=standard error; VPC=variance partitioning coefficient
[1]: Hypertension: N=29,723; Diabetes: N= 30,104; Obesity: N=30,110
[2]: Hypertension: N=29,594; Diabetes: N= 30,067; Obesity: N=9,048

© 2023 Ko S et al. *JAMA Network Open.*

eTable 9. Geographic variance partitioning for CVD risk factors along with variance and standard errors among middle-aged individuals, stratified by sex.

	Hypertension		Diabetes		Obesity[1]	
	Var (SE)	VPC (%)	Var (SE)	VPC (%)	Var (SE)	VPC (%)
**Women**						
State	0.099 (0.028)	27.9	0.335 (0.091)	48.1	0.525 (0.144)	43.1
District	0.047 (0.004)	13.2	0.118 (0.010)	17	0.232 (0.018)	19.1
Small area[2]	0.210 (0.007)	58.9	0.244 (0.015)	35	0.460 (0.026)	37.8
**Men**						
State	0.143 (0.039)	28.3	0.346 (0.094)	44.7	0.345 (0.104)	73.6
District	0.066 (0.005)	13	0.125 (0.011)	16.2	0.123 (0.038)	26.3
Small area[3]	0.297 (0.009)	58.7	0.303 (0.021)	39.2	0.0004 (0.0001)	0.1

Note. Var=variance; SE=standard error; VPC=variance partitioning coefficient
[1]: Women: age 40-49 years; Men: age 40-54 years
[2]: Hypertension: N=29,747; Diabetes: N= 30,074; Obesity: N=29,870
[3]: Hypertension: N=29,630; Diabetes: N= 30,036; Obesity: N=8,661

© 2023 Ko S et al. *JAMA Network Open.*

eTable 10. Geographic variance partitioning for CVD risk factors along with variance and standard errors among older adults, stratified by sex.

	**Hypertension**		**Diabetes**	
	Var (SE)	VPC (%)	Var (SE)	VPC (%)
**Women**				
State	0.161 (0.046)	35.3	0.500 (0.138)	51.6
District	0.060 (0.005)	13.1	0.154 (0.014)	15.9
Small area[1]	0.236 (0.010)	51.6	0.315 (0.020)	32.5
**Men**				
State	0.149 (0.042)	29.9	0.421 (0.114)	48.5
District	0.072 (0.006)	14.5	0.125 (0.012)	14.4
Small area[2]	0.277 (0.011)	55.6	0.322 (0.019)	37.1

Note. Var=variance; SE=standard error; VPC=variance partitioning coefficient
[1]: Hypertension: N=28,596; Diabetes: N= 29,062
[2]: Hypertension: N=29,778; Diabetes: N= 29,317

© 2023 Ko S et al. *JAMA Network Open.*

eTable 11. Distribution of districts based on the district-wide mean and within district variability of CVD risk factors among younger individuals, stratified by sex.

	Standard Deviation		
Mean	Low	Medium	High
**Women**			
**Hypertension**			
Low	188	42	6
Medium	47	145	44
High	1	49	185
**Diabetes**			
Low	202	34	0
Medium	34	175	27
High	0	27	208
**Obesity**			
Low	202	34	0
Medium	34	175	27
High	0	27	208
**Men**			
**Hypertension**			
Low	184	50	2
Medium	51	131	54
High	1	55	179
**Diabetes**			
Low	207	29	0
Medium	29	186	21
High	0	21	214
**Obesity**			
Low	198	37	0
Medium	35	150	51
High	3	48	184

© 2023 Ko S et al. *JAMA Network Open.*

eTable 12. Distribution of districts based on the district-wide mean and within district variability of CVD risk factors among middle-aged individuals, stratified by sex.

	Standard Deviation		
Mean	Low	Medium	High
**Women**			
**Hypertension**			
Low	169	51	16
Medium	54	105	77
High	13	80	142
**Diabetes**			
Low	211	25	0
Medium	25	191	20
High	0	20	215
**Obesity**			
Low	213	23	0
Medium	23	191	22
High	0	22	213
**Men**			
**Hypertension**			
Low	161	56	19
Medium	53	105	78
High	22	75	138
**Diabetes**			
Low	208	26	2
Medium	28	182	26
High	0	28	207
**Obesity**			
Low	199	37	0
Medium	37	165	33
High	0	33	202

© 2023 Ko S et al. *JAMA Network Open.*

eTable 13. Distribution of districts based on the district-wide mean and within district variability of CVD risk factors among older adults, stratified by sex.

	Standard Deviation		
Mean	Low	Medium	High
**Women**			
**Hypertension**			
Low	70	84	82
Medium	72	75	89
High	94	77	64
**Diabetes**			
Low	215	21	0
Medium	21	192	23
High	0	23	212
**Men**			
**Hypertension**			
Low	98	79	59
Medium	58	74	104
High	80	83	72
**Diabetes**			
Low	209	27	0
Medium	27	184	25
High	0	25	210

© 2023 Ko S et al. *JAMA Network Open.*

eTable 14. Geographic variance partitioning, stratified by sex and place of residence.

	VPC (%)					
	**Hypertension**		**Diabetes**		**Obesity**	
	Urban	Rural	Urban	Rural	Urban	Rural
**Women**						
State	24.9	31.3	55	54.9	43.7	55.6
District	14.9	15	12.4	15.7	18	17.9
Small area	60.1	53.7	32.6	29.4	38.3	26.5
**Men**						
State	23.5	31.2	42.9	48.3	23.8	44.1
District	14.6	14.2	16.2	16.6	0.8	7.1
Small area	61.9	54.6	40.8	35.1	75.4	48.7

Note. VPC=variance partitioning coefficient

© 2023 Ko S et al. *JAMA Network Open.*

eFigure 1. Flow diagram showing exclusions and final sample sizes for primary analysis of the study population.

(A) Women

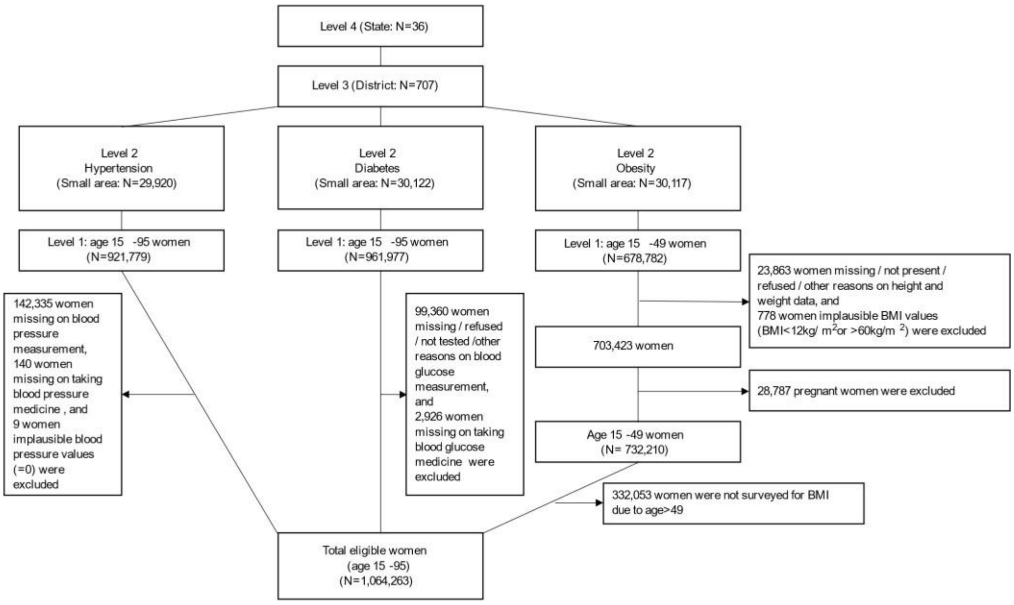

(B) Men

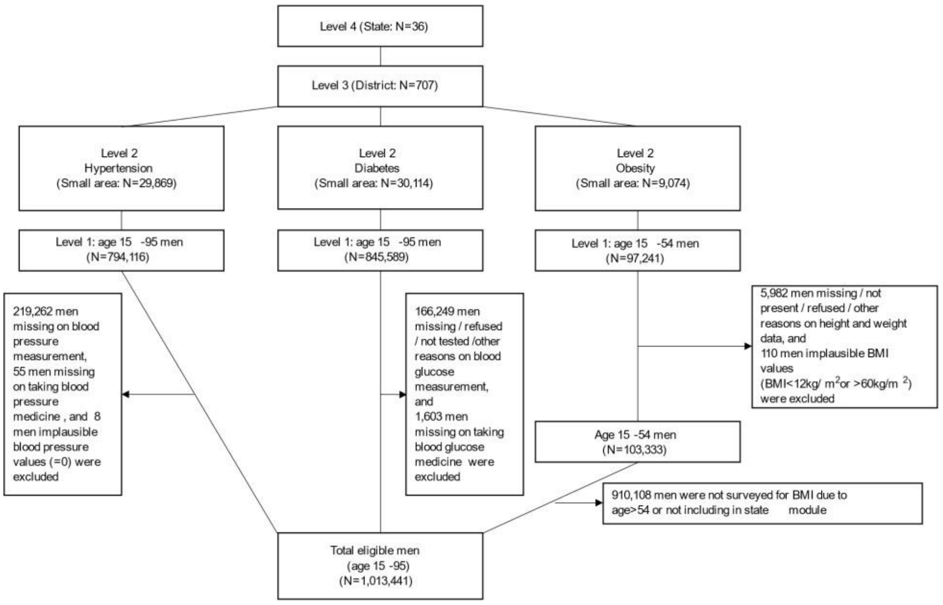

© 2023 Ko S et al. *JAMA Network Open.*

eFigure 2. Correlation between district-wide mean and within-district variability in hypertension, stratified by sex.

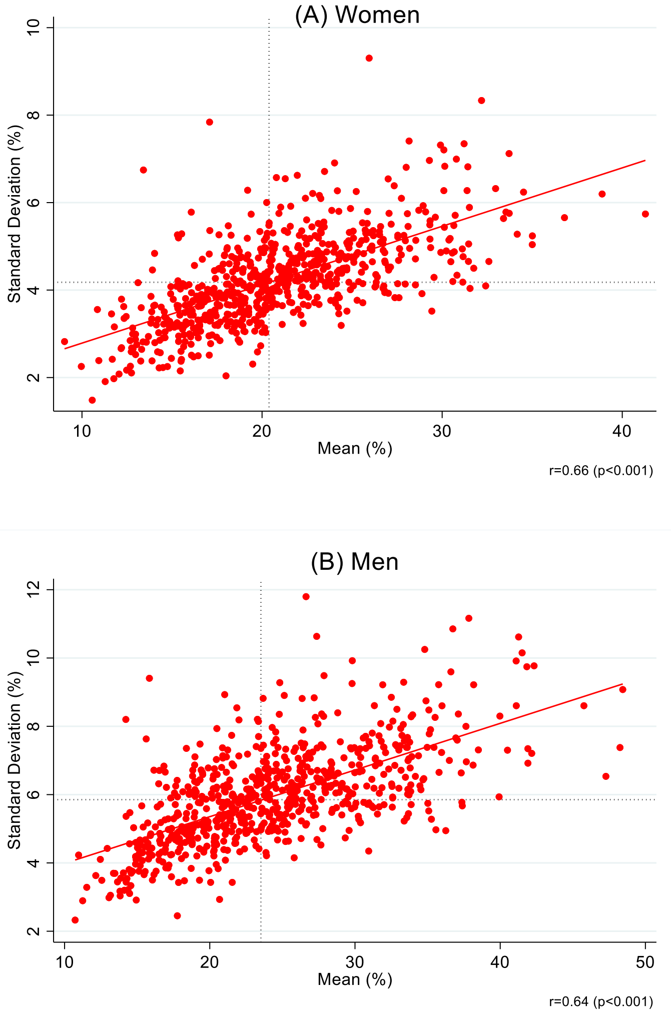

© 2023 Ko S et al. *JAMA Network Open.*

eFigure 3. Correlation between district-wide mean and within-district variability in diabetes, stratified by sex.

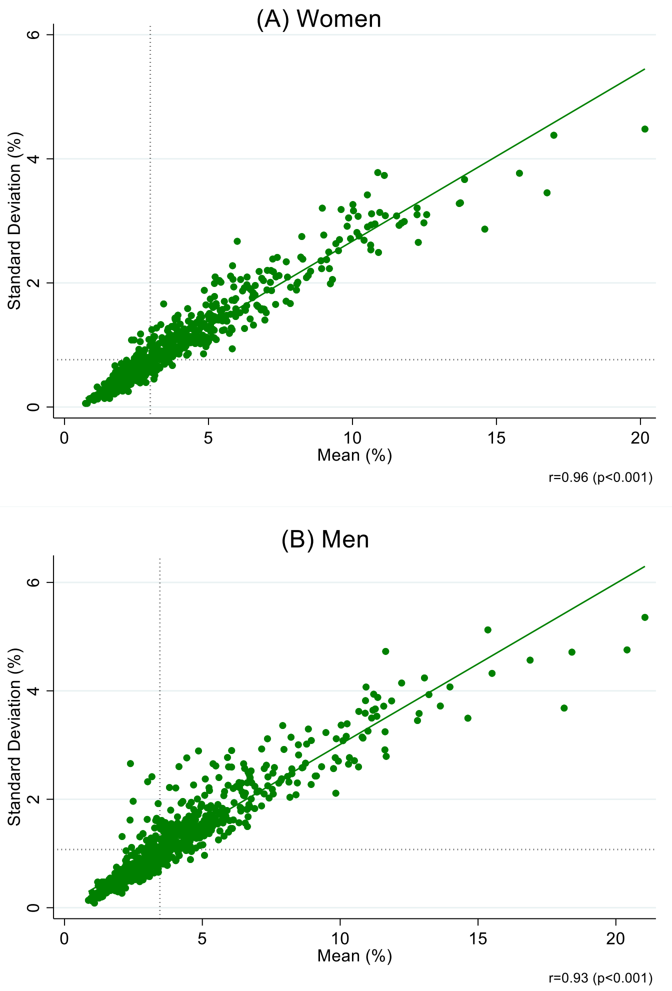

© 2023 Ko S et al. *JAMA Network Open.*

eFigure 4. Correlation between district-wide mean and within-district variability in obesity, stratified by sex.

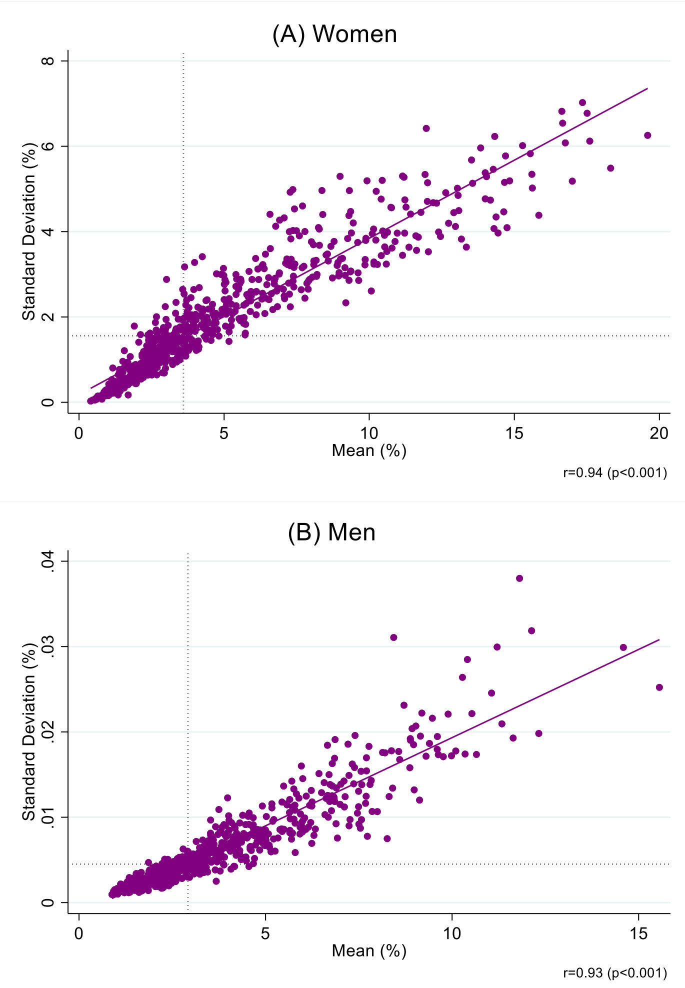

© 2023 Ko S et al. *JAMA Network Open.*

eFigure 5. District-wide mean, within-district variability, and correlation between them in hypertension among younger individuals, stratified by sex.

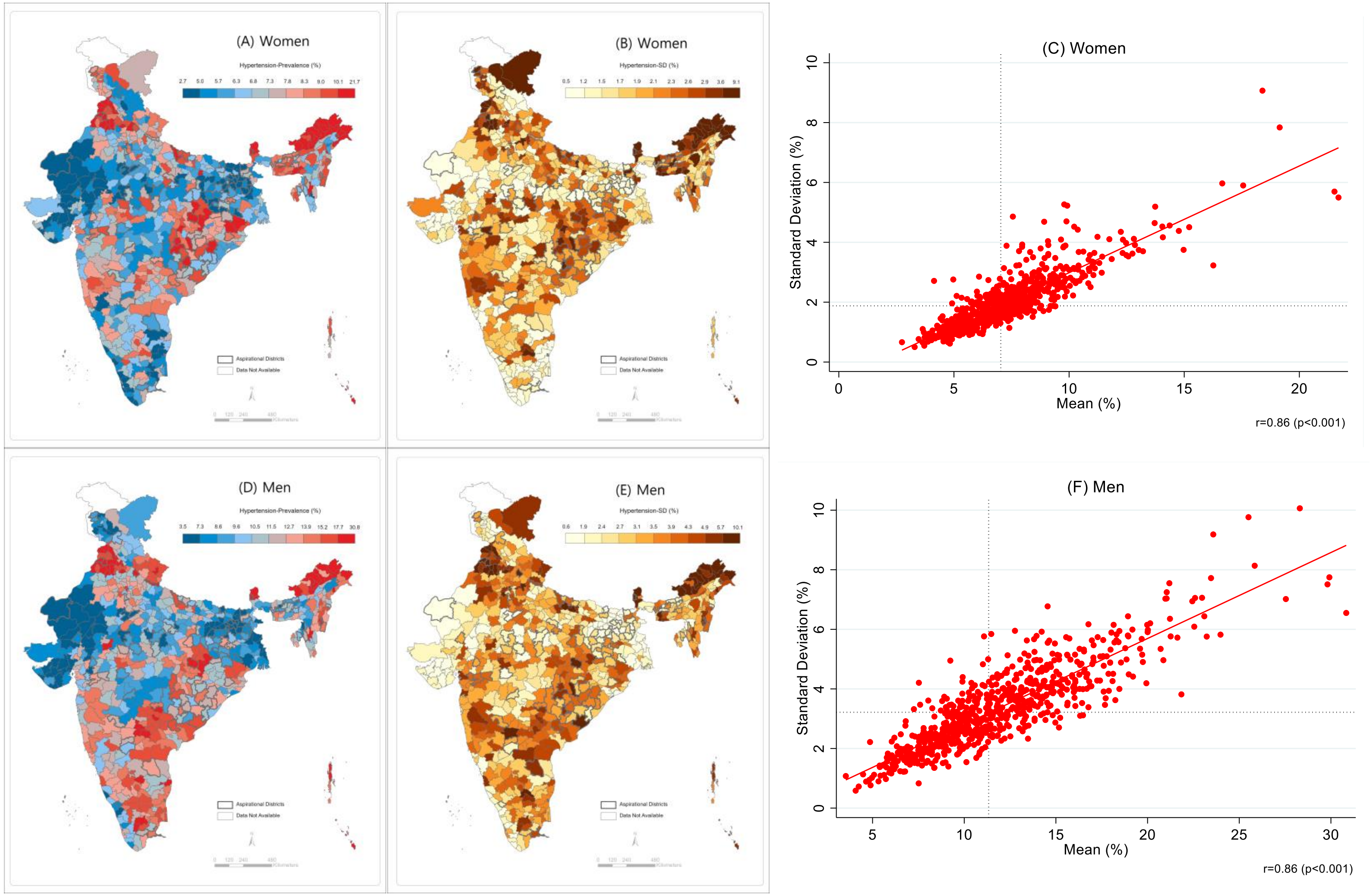

Note. SD=standard deviation

© 2023 Ko S et al. *JAMA Network Open.*

eFigure 6. District-wide mean, within-district variability, and correlation between them in hypertension among middle-aged individuals, stratified by sex.

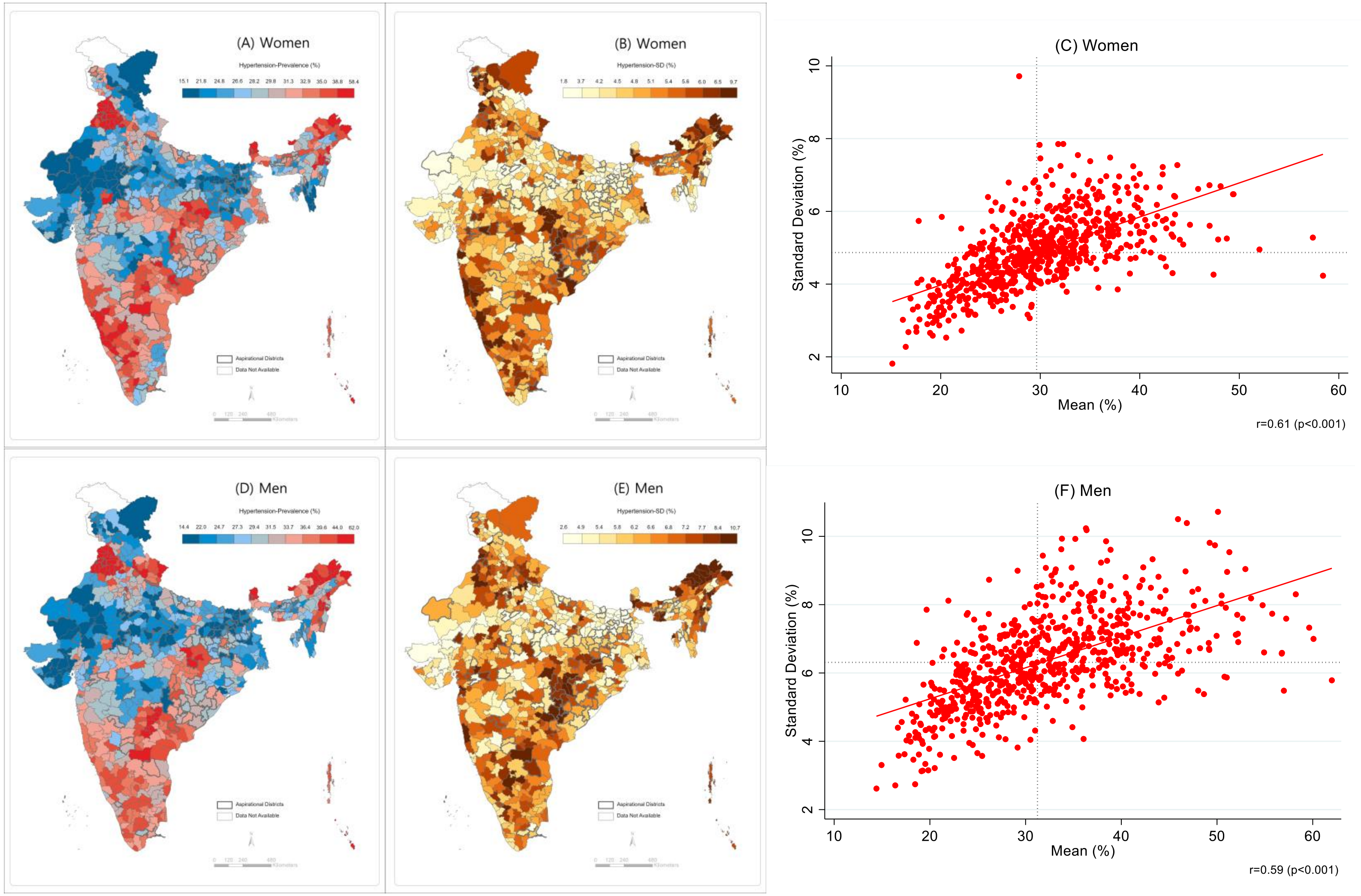

Note. SD=standard deviation

© 2023 Ko S et al. *JAMA Network Open.*

eFigure 7. District-wide mean, within-district variability, and correlation between them in hypertension among older adults, stratified by sex.

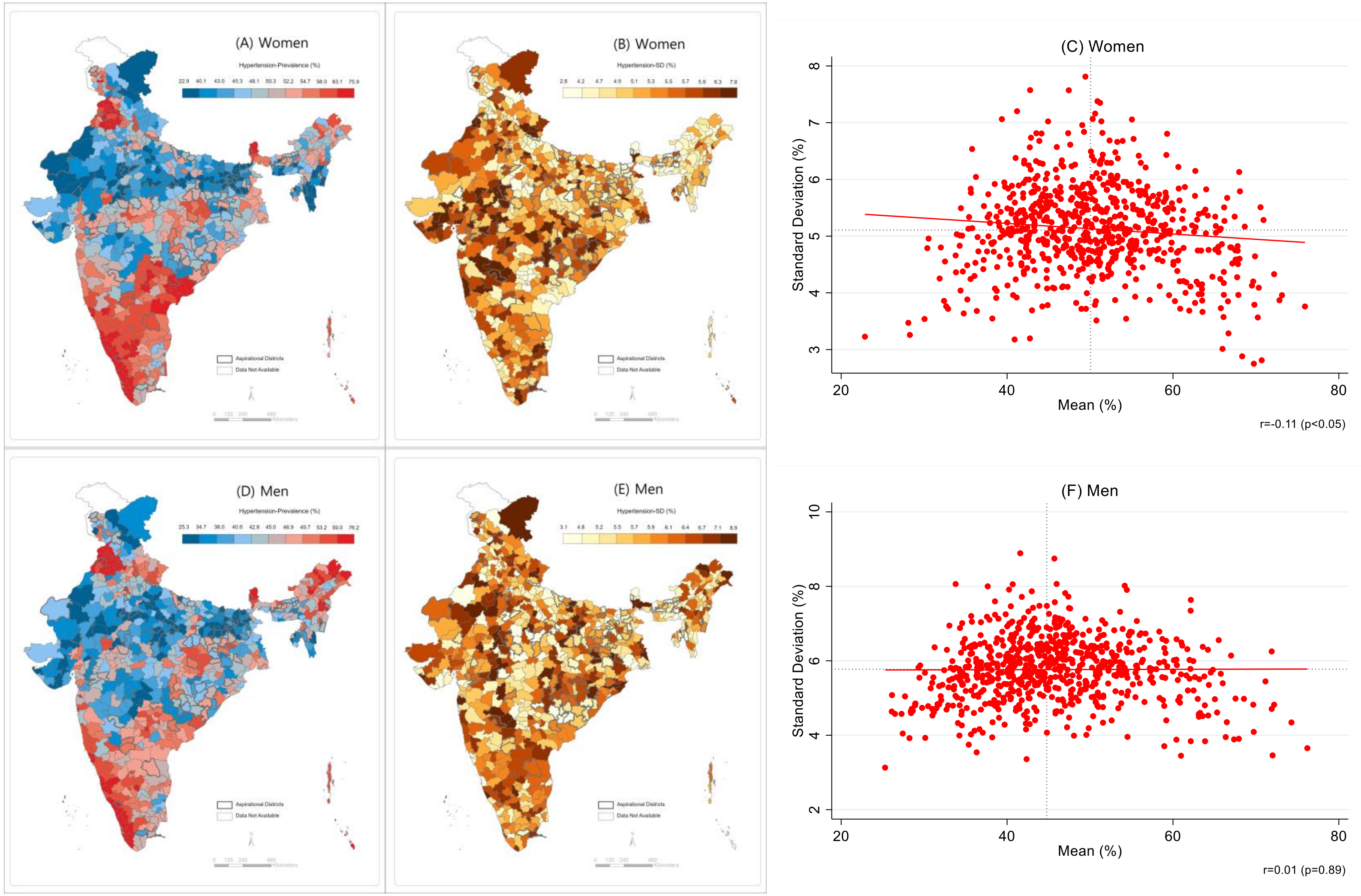

Note. SD=standard deviation

© 2023 Ko S et al. *JAMA Network Open.*

eFigure 8. District-wide mean, within-district variability, and correlation between them in diabetes among younger individuals, stratified by sex.

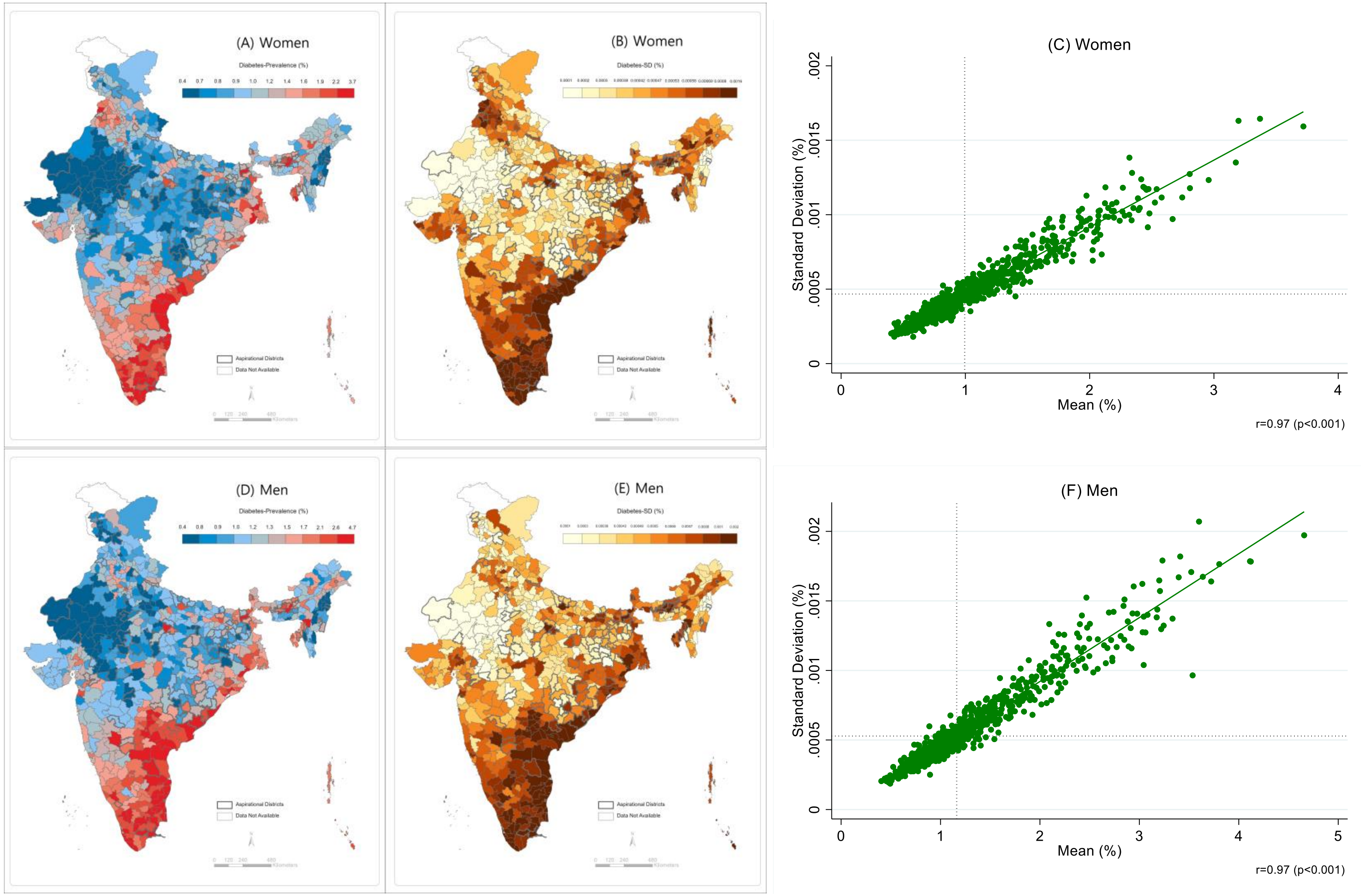

Note. SD=standard deviation

© 2023 Ko S et al. *JAMA Network Open.*

eFigure 9. District-wide mean, within-district variability, and correlation between them in diabetes among middle-aged individuals, stratified by sex.

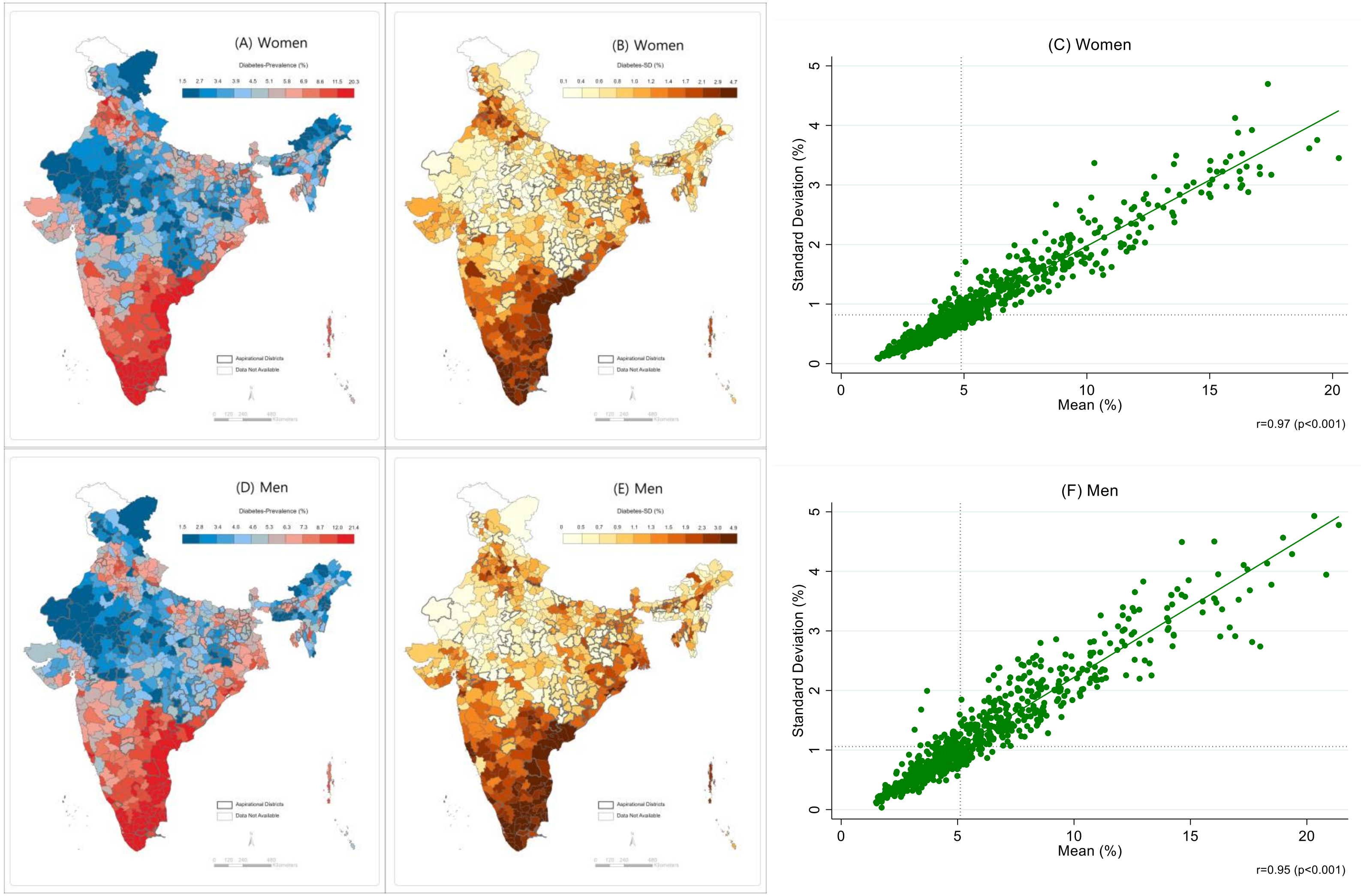

Note. SD=standard deviation

© 2023 Ko S et al. *JAMA Network Open.*

eFigure 10. District-wide mean, within-district variability, and correlation between them in diabetes among older adults, stratified by sex.

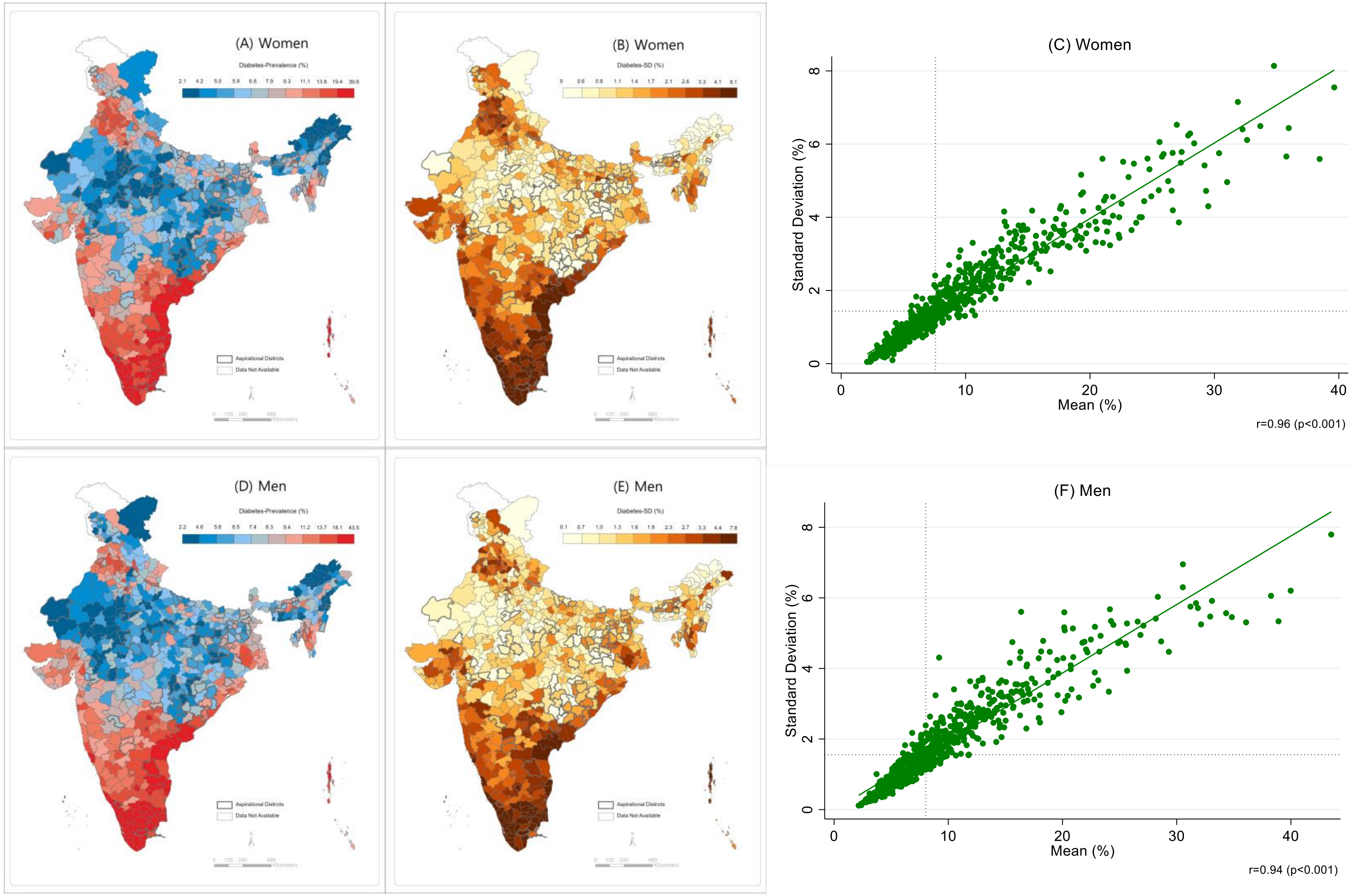

Note. SD=standard deviation

© 2023 Ko S et al. *JAMA Network Open.*

eFigure 11. District-wide mean, within-district variability, and correlation between them in obesity among younger individuals, stratified by sex.

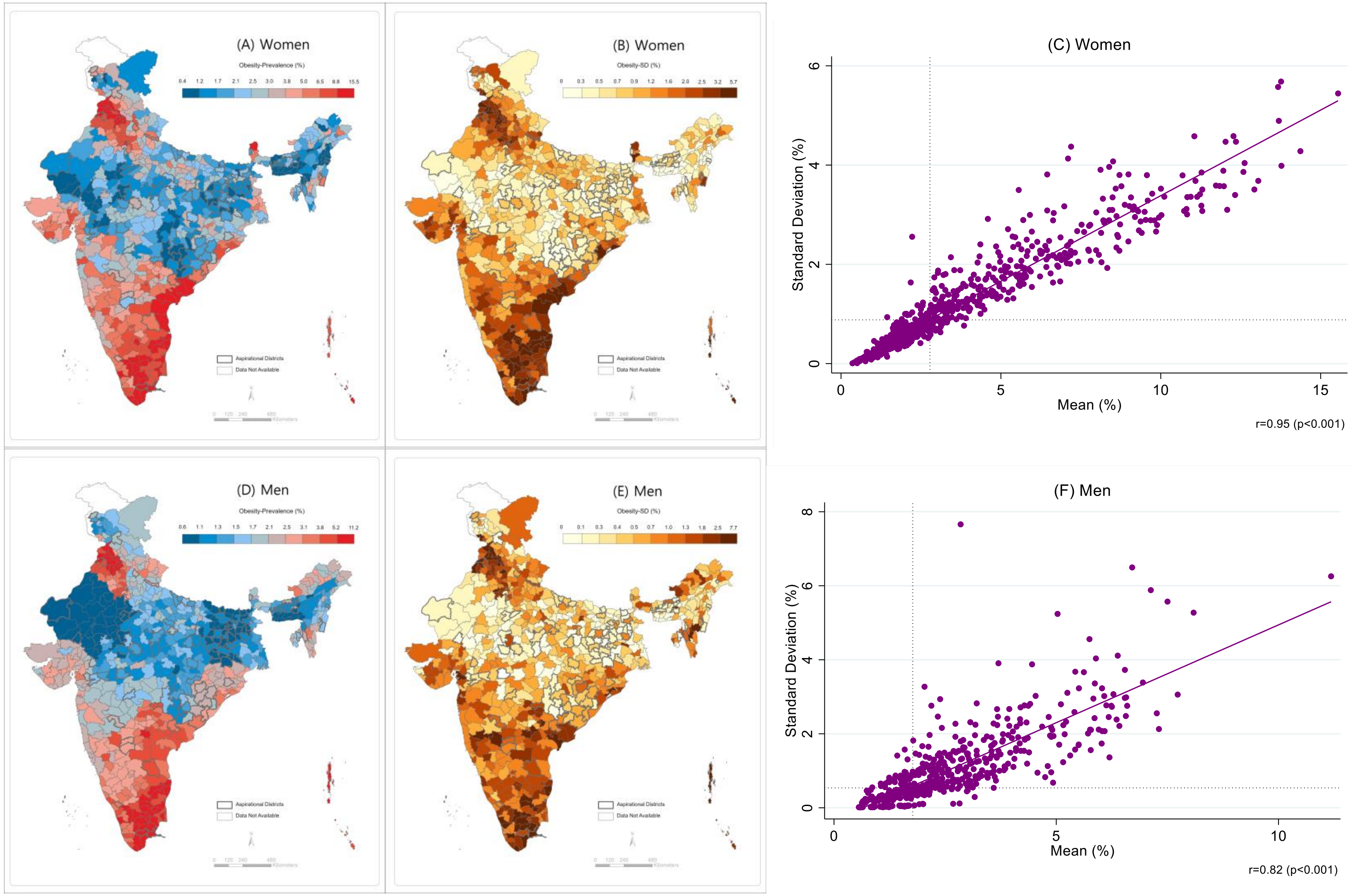

Note. SD=standard deviation

© 2023 Ko S et al. *JAMA Network Open.*

eFigure 12. District-wide mean, within-district variability, and correlation between them in obesity among middle-aged individuals, stratified by sex.

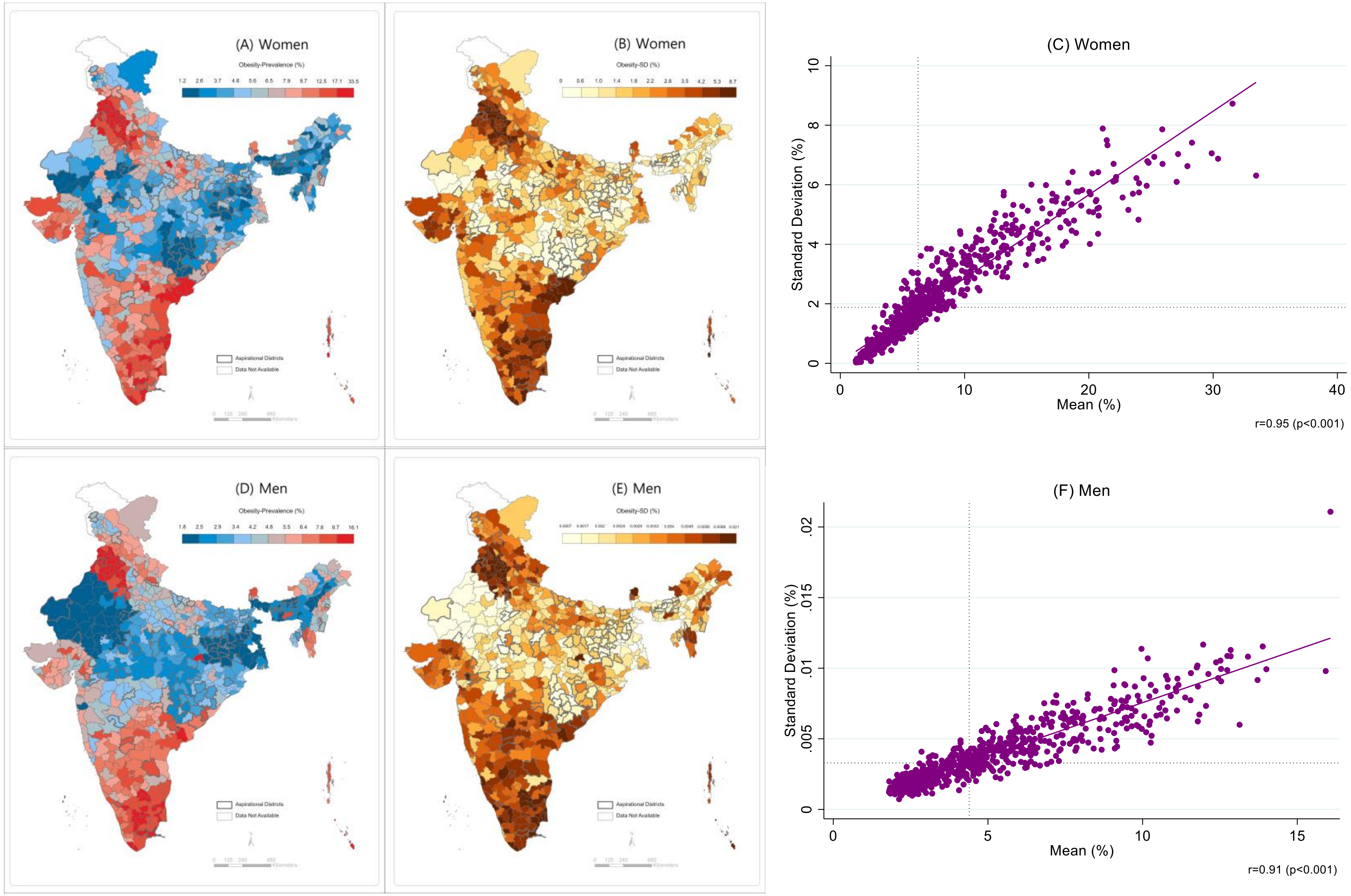

Note. SD=standard deviation

© 2023 Ko S et al. *JAMA Network Open.*
