# Supplementary material for: Small Area Geographic Estimates of Cardiovascular Disease Risk Factors in India
Source: JAMA Netw Open. 2023 Oct 12;6(10):e2337171. doi: 10.1001/jamanetworkopen.2023.37171 (PMC10570875; doi:10.1001/jamanetworkopen.2023.37171)
Supplement: Supplement 2. — Data Sharing Statement [file jamanetwopen-e2337171-s002.pdf]

Data Sharing Statement

Ko. Small Area Geographic Estimates of Cardiovascular Disease Risk Factors in India. *JAMA Netw Open*. Published October 12, 2023. doi:10.1001/jamanetworkopen.2023.37171

Data
Data available: No

Additional Information
Explanation for why data not available: The study is based on publicly available data and can be accessed from https://dhsprogram.com/data/available-datasets.cfm.
